# The detection paradox: How COVID-19 surveillance patterns shifted from lower-income to more affluent neighborhoods in Miami-Dade County

**DOI:** 10.1371/journal.pone.0361211

**Published:** 2026-10-23

**Authors:** Levente Juhász, Nana Aisha Garba, Imelda K. Moise, Prasad B. Bhoite, Christopher Clark, Rachel D. Clarke, Nicole Kikuchi Stuver, David R. Brown

**Affiliations:** 1 Fort Lauderdale Research and Education Center, School of Forest, Fisheries and Geomatics Sciences, University of Florida, Davie, Florida, United States of America; 2 Department of Medical Education, Herbert Wertheim College of Medicine, Florida International University, Miami, Florida, United States of America; 3 Department of Medical Education, Dr. Kiran C. Patel College of Allopathic Medicine (NSU MD), Nova Southeastern University, Fort Lauderdale, Florida, United States of America; 4 Office of Clinical Affairs, Herbert Wertheim College of Medicine, Florida International University, Miami, Florida, United States of America; 5 Lee Health, Fort Myers, Florida, United States of America; 6 Bureau of Epidemiology, Florida Department of Health, Tallahassee, Florida, United States of America; 7 Department of Family Medicine and Community Health, Herbert Wertheim College of Medicine, Florida International University, Miami, Florida, United States of America; Instituto Nacional de Salud Publica Centro de Investigaciones sobre Enfermedades Infecciosas, MEXICO

## Abstract

Passive disease surveillance systems may systematically undercount burden in low-resource minority neighborhoods, yet fine-scale evidence of this phenomenon remains limited. We examined spatiotemporal patterns of reported COVID-19 cases across Miami-Dade County's diverse neighborhoods and investigated which socioeconomic and demographic characteristics predicted areas of elevated and suppressed case rates. In an ecological, cross-sectional analysis of 491,769 geocoded cases across 1,831 census block groups from March 2020 to May 2021, we applied negative binomial regression with spatial eigenvector filtering and Getis-Ord Gi* hot spot analysis across five pandemic periods aligned with major policy shifts.

The analysis revealed a detection paradox. Early hot spots concentrated in lower-income census block groups (median household income: $52,022–$55,676), but by September 2020, hot spots had shifted to more affluent areas (median: $77,069). Historically Black neighborhoods along the I-95 corridor emerged as persistent cold spots despite multiple structural risk factors: lower incomes, reduced insurance coverage, and limited healthcare access. From Period 3 onward, predominantly Black census block groups showed significantly lower reported rates (incidence rate ratio [IRR]: 0.991–0.995, p < 0.001). Household overcrowding was tested but retained in no final model.

These paradoxical patterns suggest that as testing expanded, surveillance increasingly tracked healthcare utilization rather than transmission. What appeared as neighborhood “protection” likely reflected barriers to testing access shaped by transportation constraints, work schedule inflexibility, and historical relationships with medical institutions. Census block group analysis revealed these dynamics obscured at broader geographic scales.

The findings challenge the assumption that reported case rates approximate true disease burden and have direct implications for emergency response planning: surveillance systems require complementary detection streams, risk-adjusted monitoring for underdetection, and targeted outreach where poverty and limited access to testing are concentrated but reported rates are low.

## Introduction

The COVID-19 pandemic generated striking geographic variations in disease burden [1–6]. While viral transmission follows biological principles [7], certain populations faced elevated risks. Frontline workers, residents of economically constrained areas, and demographic groups experienced disproportionate rates of infection, hospitalization, and mortality [8,9]. These patterns emerged from intersecting factors: baseline population characteristics, variable exposure risks, and asymmetric access to protective resources including testing and treatment [10].

Transportation and mobility shaped exposure early in the pandemic, as transit and air travel accelerated spread while the ability to reduce movement fell along demographic and economic lines [11–20]. The same mobility differences shaped access to testing, so their imprint on reported cases may reflect detection as much as transmission.

Beyond transmission, healthcare infrastructure shaped observed disease patterns. COVID-19 testing varied by geography, socioeconomic status, and demographic characteristics [21–23]. This patchwork of access poses a challenge: do reported case rates capture actual disease burden, or merely map the contours of our detection systems [21]?

Geographic analyses are essential for understanding COVID-19 variations. Census block group (CBG)-level factors such as housing density, essential worker concentrations, and healthcare accessibility create distinct risk patterns [19,21,24–26]. Fine-scale spatial analyses identified hot spots and cold spots that broader units mask while capturing how community-level factors shape disease detection [18,21,25,27]. Temporal analyses further revealed how spatial patterns evolve with policy changes, testing expansion, and behavioral adaptations [27–30].

Miami-Dade County, with its demographically diverse population and spatial segregation from transit-dependent urban cores to car-dependent suburbs, provides an ideal setting for examining these dynamics. Previous ZIP code-level research found substantial COVID-19 variation linked to socioeconomic factors [1,31].

Most COVID-19 analyses in this region have relied on ZIP code tabulation areas, which average over neighborhoods that differ sharply in income, race, and access to care. Building on that work, we argue that the census block group is the more appropriate unit for surveillance and resource allocation, because it resolves the within-ZIP heterogeneity that shapes both transmission and detection. Demonstrating this resolution and showing what it reveals about who is counted during a pandemic is a central aim of this study.

This study examines the spatiotemporal distribution of reported COVID-19 cases across Miami-Dade County's urban CBGs from March 2020 to May 2021, grouped into five distinct three-month periods that align with major policy shifts from initial lockdowns through vaccine rollout [32]. Using spatial statistical methods including hot spot analysis and regression modeling with spatial eigenvector filtering, we investigate: (1) How reported case patterns evolved across CBGs over time; (2) Which socioeconomic and demographic factors predicted spatial clustering of high and low case rates; and (3) How unmeasured spatial factors might explain observed patterns. Answering these questions at the CBG level reveals localized dynamics to inform pandemic preparedness.

## Methods

This study is ecological. The unit of analysis is the census block group rather than the individual, and no reporting guideline covers this design directly. We followed the STROBE guideline for cross-sectional studies where its items apply, and the completed checklist marks the items that do not (S1 Checklist).

### Study setting

The study location of Miami-Dade County, situated in Southeast Florida, is part of the Miami-Fort Lauderdale-West Palm Beach Metropolitan Statistical Area (MSA), the eighth largest MSA in the United States as of the 2020 decennial census. The 2017–2021 American Community Survey (ACS) estimates, which encompass our entire study period, report a population of approximately 2.7 million [33], which renders Miami-Dade County the seventh-most populous county in the nation, making it a critical region for studying urban health dynamics. The county is highly racially and ethnically diverse, with 68.5% of residents identifying as Hispanic or Latino, 17.7% as Black or African American, and 13.3% as White (non-Hispanic) [33]. Additionally, 54.0% of the population is foreign-born [34], reflecting the county’s substantial immigrant presence. The 2017–2021 ACS estimated a median household income of $57,815, with 15.7% of residents living below the poverty line [35]. Housing density varies significantly, with 2.83 persons per household on average [36], yet overcrowding remains a challenge in lower-income neighborhoods where multi-generational households and high rental costs contribute to increased population density. The median gross rent of $1,470 [36] represents 30.5% of median household income [35], exceeding the 30% threshold that federal standards define as housing cost-burdened [37]. In fact, 53.7% of occupied units spent 35% or more of their household income on rent. This burden is even greater for households below the poverty line, where housing costs can consume 50% or more of income, contributing directly to the overcrowding patterns observed in lower-income neighborhoods as families consolidate housing to reduce per-capita costs. Healthcare accessibility is also a concern, with 15.6% of residents under age 65 lacking health insurance, and areas with lower median incomes experiencing higher rates of preventable hospitalizations due to transportation barriers, language obstacles, and fewer healthcare providers [38].

Our analysis focuses on CBGs within the Urban Development Boundary (UDB) to maintain a consistent urban context for assessing CBG-level COVID-19 patterns (S1a Fig). Focusing on densely populated regions within the UDB minimizes analytical distortions from sparsely populated areas, ensuring findings reflect urban health conditions and support targeted interventions. The analysis includes 1,831 CBGs within the UDB, covering 2.57 million residents (over 95% of the county's total population) according to 2017–2021 ACS 5-year estimates.

The timeframe for this study extended from March 2020 to May 2021. This period covered a total of 15 months, chosen to align with the significant early waves and evolving dynamics of the COVID-19 pandemic. To enable a granular analysis of temporal shifts, the entire 15-month duration was divided into five distinct, consecutive 3-month study periods. This segmentation captures variations in pandemic-related patterns and public health responses as the situation unfolded.

### Data sources

This study integrates multiple data sources to provide a comprehensive analysis of COVID-19 patterns and their associations with CBG characteristics in Miami-Dade County. Demographic, socioeconomic, housing, and healthcare access data were obtained from the U.S. Census Bureau. To assess the spatial patterns of localized infection rates and disease burden, COVID-19 case data were sourced from the Florida Department of Health (FDOH).

#### COVID case data.

COVID-19 case data were obtained from the Florida Department of Health's (FDOH) Merlin surveillance system [39], retaining only addresses (residence if available, testing site if residence unavailable, or laboratory if neither was available) and infection dates from March 2020 to May 2021 for geocoding and aggregation at the CBG level. The delivered records carried a single address field with no indicator of which source had been used, so residential and testing site addresses could not be distinguished by flag. Inspection of the geocoded points was consistent with residential addresses throughout. Data were accessed for research purposes on November 9, 2022.

#### Community-level population and household characteristics.

Population and household data were obtained from the U.S. Census Bureau American Community Survey (ACS) 2017–2021 five-year estimates at the CBG level to capture fine-scale spatial heterogeneity, particularly important in Miami-Dade County given its complex segregation patterns and diverse population [40–42].

### Data processing

#### Geocoding and aggregation.

To analyze the spatial distribution of cases, individual patient home addresses were geocoded using the ESRI ArcGIS REST Geocoding service and projected to NAD83/UTM Zone 17N (EPSG:26917) coordinate system. Cases were then aggregated at the CBG level within the Miami-Dade County UDB. S1b Fig illustrates the spatial distribution of cases using proportional dot density mapping to preserve patient anonymity while conveying disease distribution.

A total of 520,223 COVID-19 case records were available for analysis. Of these, 519,931 (99.9%) were successfully geocoded to a physical address. The remaining 292 cases had incomplete, invalid (e.g., “Homeless, Miami”), or foreign addresses. These were excluded because geographic coordinates could not be assigned. Of the geocoded cases, 491,769 met the spatial and temporal inclusion criteria, having occurred between March 2020 and May 2021 within CBGs located inside the Miami-Dade County Urban Development Boundary (UDB). Cases falling outside these parameters were excluded from further analysis. Because the address field did not identify its source, we examined points where multiple cases shared an address to rule out clustering at testing or laboratory sites. Overlapping address points were attributed to high-occupancy residential and institutional sites such as jails, nursing homes, and large apartment complexes, and did not reflect centralized testing or laboratory locations, thereby indicating genuine clustering at the CBG level.

#### Rate calculation.

The total number of COVID-19 infections per CBG was used to calculate infection rates per 1,000 people, ensuring comparability across different population densities. Despite being within the UDB, CBGs can vary significantly in population size, event counts, and spatial heterogeneity, making it challenging to detect true patterns without statistical adjustments. To account for rate instability in sparsely populated block groups, we applied Empirical Bayes (EB) smoothing to the raw infection rates. This approach adjusts observed rates by shrinking them toward the overall mean, reducing the influence of random fluctuations in low-populated areas, which is a common issue in large block groups with small resident counts [43,44].

Smoothed infection rates were computed using the *spdep* package in R [45], in-line with established spatial epidemiological techniques to produce more stable and interpretable rate maps. This technique enhances the reliability of CBG comparisons. While our primary application of EB smoothing is spatial, the broader framework has also been widely employed in epidemiology to balance local precision with broader epidemiological trends [46] and to forecast temporal epidemic patterns [47].

### Policy context

The study period encompassed five distinct policy phases (Table 1; detailed timeline in Supporting Information S1 Table). Period 1: severe testing limitations (300 daily tests, > 20% positivity) with stay-at-home orders [32,48–50]. Period 2: summer surge prompting curfews despite expanded testing (16,000 + daily) [32,49,51]. Period 3: gradual reopening amid laboratory supply shortages [32,52,53]. Period 4: peak cases coinciding with initial vaccine rollout to elderly residents [32,49,54]. Period 5: expanded vaccination with improved testing access and mixed reopening signals [32,49,54]. These policy shifts shaped both transmission patterns and case detection across communities. These periods provide a temporal frame for organizing the spatial analysis. They are aligned with documented shifts in testing capacity and policy, but the study does not estimate the causal effect of any individual policy.

**Table 1:** Key policy context by study period in Miami-Dade County.

Study Period	Testing Availability	Major Restrictions	Vaccine Status	Key Context
1 (March - May 2020)	• Severely limited • 300 tests daily • 20% positivity rate • Appointment only	• Stay-at-home orders • Non-essential businesses closed • Parks/beaches closed • Excluded from state reopening	• Not available	• Initial outbreak • Testing scarcity • Indoor mask mandate
2 (June - Aug 2020)	• Under-resourced • Some walk-up sites • Long lines forming • High positivity persists	• Curfew (10pm) • Indoor dining banned (July) • Entertainment venues re-closed • Masks required in public	• Not available	• Summer surge • 25% of FL cases
3 (September - November 2020)	• Supply constrained • Test kit shortages • Limited lab capacity • Appointments required	• Gradual reopening • Curfew relaxed • Most businesses reopened • Mask enforcement ended	• Not available	• State final recovery phase • Schools reopened (October)
4 (December - February 2021)	• Surge overwhelmed • Winter case surge • Holiday testing demand • System strained	• Curfew continued • Most businesses operated with restrictions	• Limited to 65+ and healthcare	• Holiday surge • Vaccine scarcity
5 (March - May 2021)	• Moderately accessible • Multiple walk-in sites • Demand decreased	• Curfew lifted (April) • Some restrictions restored • Emergency orders extended weekly	• Available to all adults	• Vaccine expansion • Mixed reopening signals • Policy uncertainty

### Outcome measures

The study assessed three primary outcome measures at the CBG level for each of the 3-month study periods from March 2020 to May 2021. These were:

1. **Case counts**: The total number of reported positive test results per CBG. These counts were the dependent variable in Negative Binomial Regression models, with population included as an offset to allow for rate interpretation.
2. **EB smoothed infection frequencie**s: The infection rate per 1,000 people for each CBG, smoothed using EB for rate stability. These were used in the Getis-Ord *G*_i_* analysis to identify hot and cold spots.
3. **Spatial cluster membership**: A categorical variable (hot spot, cold spot, or non-significant) based on the Getis-Ord *G*_i_* analysis. This was used as the dependent variable in logistic regression models to predict cluster membership.

### Census block group (CBG) socioeconomic measures

S2 Table presents the comprehensive set of 39 community level variables selected for analysis, organized into ten thematic categories: race, ethnicity, age structure, educational attainment, income, housing characteristics, healthcare access, employment status, transportation modes, and household structure/linguistic characteristics. These variables were selected from the several hundred available ACS block group indicators based on their established relevance to COVID-19 transmission risk, exposure opportunities, and healthcare access barriers as documented in prior literature [1,10,31,55]. The selection process prioritized variables that capture: (1) demographic composition affecting disease susceptibility and transmission networks; (2) socioeconomic factors influencing ability to socially distance and access healthcare; (3) household and housing characteristics affecting within-home transmission risk; (4) transportation patterns determining exposure during commutes; and (5) linguistic and cultural factors potentially affecting information access and healthcare utilization. Together, these measures provide a comprehensive profile of CBG-level factors that may influence COVID-19 transmission and detection patterns. While a total of 39 variables were selected for analysis, the 36 measures listed individually in S2 Table were included in the statistical models. For variable sets that are compositional (i.e., race, age, education), one category was excluded to serve as a reference group. This approach prevents multicollinearity in the models and ensures that the effects of specific population subgroups are interpreted relative to the defined baseline (i.e., the reference variable). This allows for a meaningful interpretation of each variable’s association with COVID-19 rates. For example, the proportions of age groups under 5, 5–17, and 65 or older are compared against the proportions of the adult population aged 18–64.

### Geospatial analysis

The first part of this study employed geospatial methods to analyze the spatial distribution of COVID-19 infections across Miami-Dade County, integrating hot spot analysis and spatial regression modeling with spatial eigenvector filtering to enhance pattern detection and model precision.

#### Hot spot analysis.

To identify spatial clusters of high and low COVID-19 infection rates, we used the local Getis-Ord *G*_i_* statistic [56]. This technique allowed for the detection of localized areas of elevated disease burden, highlighting block groups where transmission risk was disproportionately high or low. The analysis was conducted in R using the *sfdep* [45] package, applying 499 permutations to assess statistical significance. We used EB smoothed rates to calculate the local *G*_i_* statistic (Eq 1 in S1 File).

We defined neighbors using the queen contiguity criterion, i.e., block groups sharing at least one boundary point were considered adjacent. The spatial weights matrix was row-standardized.

Clusters were classified as statistically significant hot spots or cold spots based on whether the *G*_i_* z-scores fell in the upper or lower tails of the null distribution generated by 499 permutations. Only clusters significant at the 5% level (i.e., *p*≤0.05) were retained. The resulting patterns highlight areas where unusually high or low COVID-19 incidence values are unlikely to have occurred by chance.

To compare the socio-demographic characteristics of CBGs within identified hot spots and cold spots to the overall county distribution for each period, non-parametric Mann-Whitney U tests were employed. This test compares two independent groups when the data is not normally distributed. For each characteristic listed, the values for block groups classified as hot spots were compared against the countywide distribution. A separate comparison was made for cold spot block groups.

#### Spatial regression modeling with spatial eigenvector filtering.

To model COVID-19 case counts within CBGs, we used negative binomial regression (NBR) models to account for overdispersion in the count data. The dependent variable was the number of reported COVID-19 cases during a given 3-month long study period between March 2020 and May 2021. The population density was included as a control measure. To model rates of COVID-19 cases and to adjust for population size, the total population was included as a log-transformed offset in the NBR, rather than a standard covariate. The model specification is provided in S1 File (Eq 2). Median household income was unavailable for 203 block groups and reported as US$250,000 or more, the ACS top code, for 20 others. Both were treated as missing, so the regression models were fit on the 1,608 block groups with a numeric income value. The same analytic set was used for every regression model, including those in which income was not retained after variable selection. The Getis-Ord Gi* analysis used all 1,831 block groups. As a check, we recoded the 20 top-coded block groups to US$250,000 and refit the models on 1,628 block groups. We held the model specification fixed rather than repeating variable selection.

To assess residual spatial autocorrelation, we calculated the global Moran’s *I* [57] on residuals from baseline models (i.e., without spatial filters). These tests showed weak to moderate spatial autocorrelation (Moran's *I* ranging from 0.03 to 0.11, all *p* < 0.05), suggesting a need for spatial adjustment. To address this issue, we applied spatial eigenvector filtering (SEF) [58]. We computed spatial eigenvectors through decomposing the queen contiguity-based spatial weights matrix. These eigenvectors capture latent spatial structure not explained by covariates [59]. Our sensitivity analysis indicated that including the first 15 eigenvectors substantially reduced spatial autocorrelation in residuals while maintaining model parsimony. SEFs were added to the NBR models as additional predictors and are shown in Supporting Information S2 Fig. A stepwise selection of all predictors (socio-economic, control, and spatial filter) based on the Akaike information criterion (AIC) was used to retain only the most influential predictors. Including the SEFs reduced residual spatial autocorrelation in every period, with the global Moran’s *I* falling to between 0.005 and 0.088. The final models retained key socio-economic predictors and a subset of SEFs with good fit metrics. These spatially adjusted NBR models estimate the relationship between socio-economic characteristics and COVID-19 case counts while accounting for spatial structure.

#### Classification of cluster membership.

To predict whether a CBG belonged to a Hot Spot or Cold Spot cluster, logistic regression (LR) models were employed with similar explicit SEF described in the previous section. LR is suitable for binary classification tasks, making it appropriate for distinguishing between spatial clusters of unusually high or low COVID-19 case densities and other areas. The dependent variable was a binary indicator representing whether a CBG was classified as a Hot or Cold spot based on the local *G*_i_* statistics. Independent variables included SDOH measures, spatial eigenvectors to address spatial dependence, and control variables such as a CBG’s population and land area. The model specification is provided in S1 File (Eq 3).

To ensure unbiased parameter estimates, SEF was introduced to generate spatial eigenvectors from the spatial weight matrix *w* (described above under Hot spot analysis). A stepwise variable selection approach was then applied to iteratively add or remove predictors, including control, socioeconomic, and SEF variables, based on their contribution to model fit (assessed by AIC). The final model retained only the most significant predictors and successfully reduced spatial dependence in the residuals.

### Ethics approval

This study was conducted in accordance with the Declaration of Helsinki and applicable guidelines and regulations. It was approved by the Institutional Review Boards of the Florida Department of Health (IRB number 2022-012) and Florida International University (IRB number IRB-21-0081). The analysis used de-identified secondary data. The requirement for informed consent was waived because the dataset comprised all COVID-19 cases reported to the Florida Department of Health and individual consent was not practicable.

## Results

### Descriptive statistics

We observed marked demographic and socioeconomic heterogeneity across block groups (CBGs). On average, each CBG included 1,403.9 residents (range: 0–6,281, median of 1,303). Median land area per CBG was 0.39 km^2^, with population density exhibiting variation (median: 3,365 people per km^2^; mean: 4,763 people per km^2^). The population was predominantly Hispanic/Latino with a mean proportion of 66.4% (range: 0–100%). Socioeconomic indicators revealed that 15.5% of residents lived below the federal poverty level and 14.9% lacked health insurance coverage. Housing characteristics reflected a renter-occupancy rate of 44.6%, and median household income varied widely, from $7,487 to $249,706 (median $60,364), with 20 block groups top-coded at $250,000 or more and 203 without a reported value. Notably, 22.4% of the population reported limited English proficiency. Selected socio-economic characteristics are shown in Table 2, with a full distribution of study variables provided in the Supporting Information S3 Table.

**Table 2:** Summary statistics of selected socio-economic characteristics of urban block groups in Miami-Dade County (*n =* 1,831 block groups).

Variable	Mean	Median	Min.	Max.
*Percentage of Hispanic or Latino Population*	66.4	73	0	100
*Percentage of Population Below Poverty Level*	15.5	12	0	79
*Percentage of population with undergraduate or associate degrees*	29.9	29	0	100
*Median Household Income [US$]*	70,354	60,364	7,487	249,706
*Percentage of Population with No Health Insurance Coverage*	14.9	13	0	78
*Percentage of Black or African American Population*	15.5	3	0	100
*Percentage of population aged 65 or above*	17.5	16	0	100

Monthly COVID-19 case counts varied substantially over the study period, ranging from 6,227 cases in March 2020 to a peak of 82,593 in July 2020. The average monthly count of cases was 32,785, with a median of 32,000 cases. As illustrated in the top panel of Fig 1, the temporal distribution of cases revealed two prominent surges during Periods 2 and 4, while the remaining periods were characterized by comparatively lower incidence levels.

**Fig 1:**
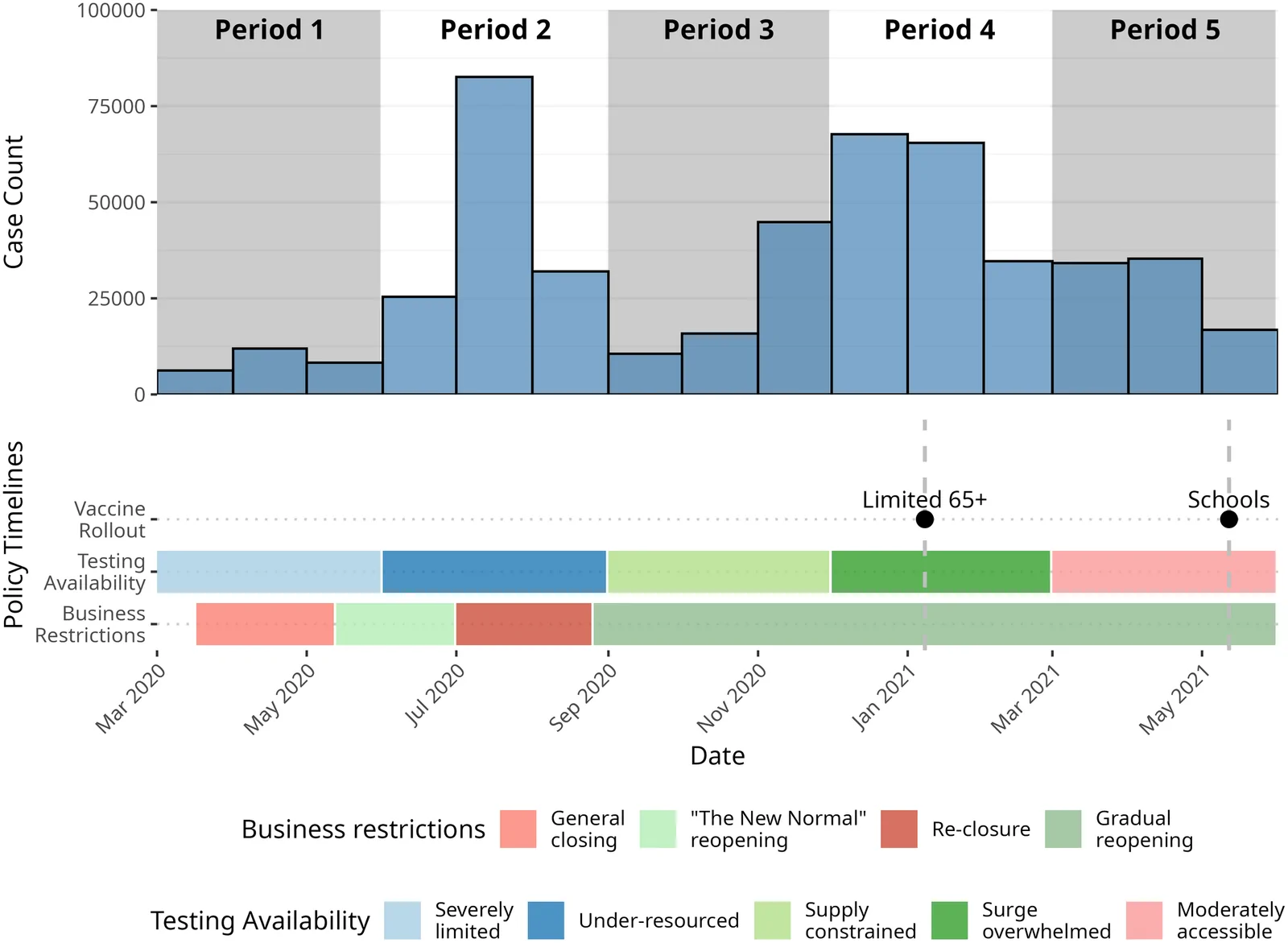
Monthly case counts in urban Miami-Dade County during the study period (top) and summary of the relevant policy context described in Table 1.

COVID-19 infection rates varied markedly across the five study periods, as summarized in Table 3. The overall rate during Period 4 (December 2020–February 2021) was approximately 6.3 times higher than that observed in Period 1 (March–May 2020), reflecting significant temporal variation in transmission intensity. In March 2020, at least one confirmed case was reported in 90.4% of CBGs. By the end of Period 1, this figure had risen to 98.9%, suggesting widespread geographic distribution of cases from the outset of the pandemic. For the remainder of the study period (Periods 2–5), all inhabited CBGs recorded at least one case. These temporal patterns provided the empirical foundation for subsequent spatial clustering and regression analyses.

**Table 3:** Summary statistics of COVID-19 cases in urban block groups in Miami-Dade County by study period. Rates are unweighted means of block group rates per 1,000 residents.

Period	Dates	Total cases	Avg. cases / CBG	Median cases/ CBG	Avg. rate [/1k]
Period 1	March 2020 – May 2020	26,422	14.4	12	11.4
Period 2	June 2020 – August 2020	139,993	76.5	64	59.2
Period 3	September 2020 – November 2020	71,265	38.9	32	31.1
Period 4	December 2020 – February 2021	167,791	91.6	77	71.5
Period 5	March 2021 – May 2021	86,298	47.1	41	37.4

### Factors associated with overall COVID-19 rates (NBR models)

To assess predictors of reported cases, negative binomial regression (NBR) models were fit separately for each period. Fig 2 presents the set of significant predictors identified in each model. Variables were classified by temporal persistence and by effect type. Persistence categories were early (Period 1 only), middle (Periods 2–3), late (Periods 4–5), and persistent (present in ≥3 periods). By effect type, *risk* factors denote positive associations with detected case rates, and *protective* factors denote negative associations. Full model estimates, including incidence rate ratios (IRRs), 95% confidence intervals, significance levels, and model diagnostics, are provided in Supporting Information S4 Table.

**Fig 2:**
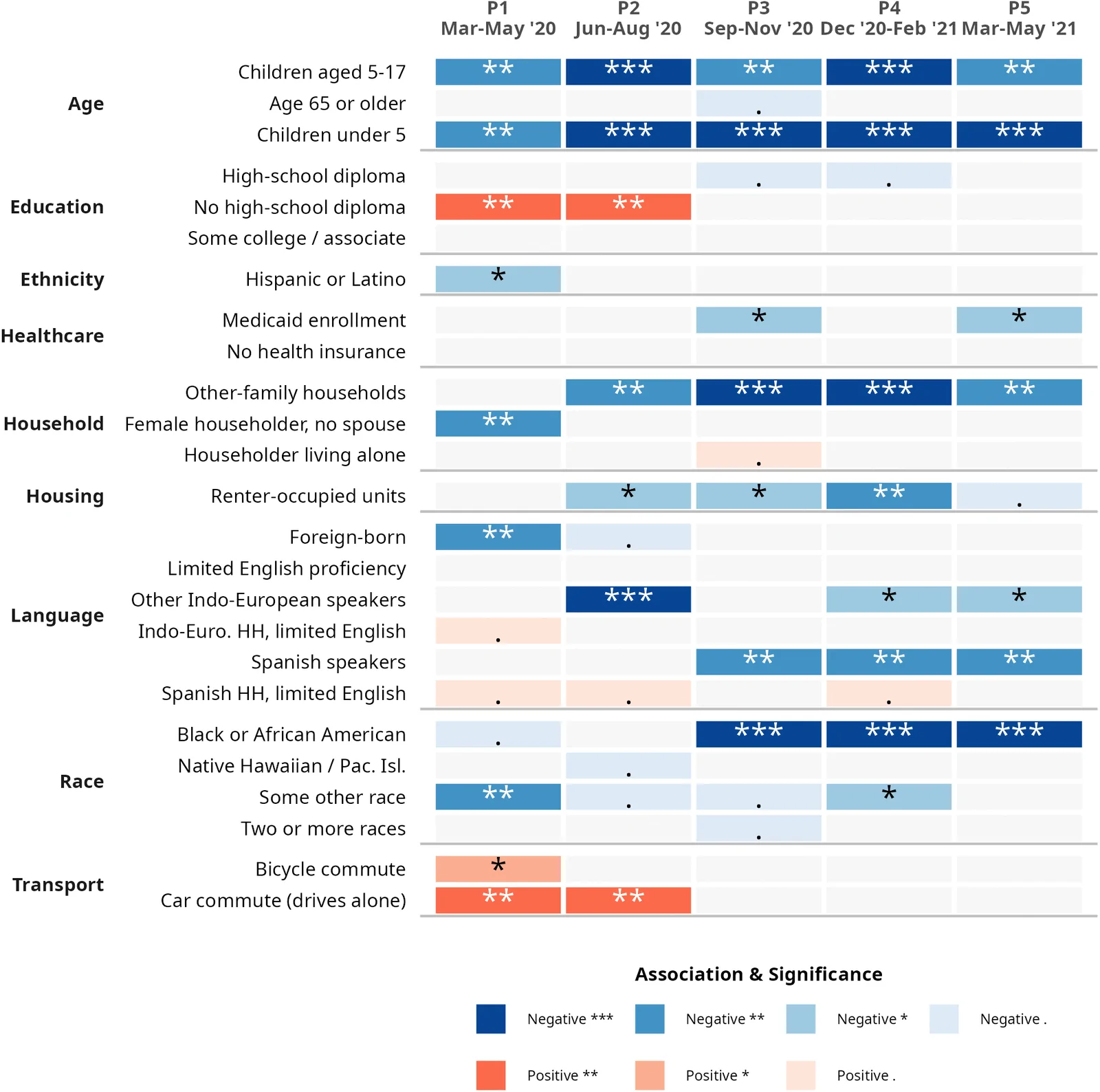
Socioeconomic predictors of reported COVID-19 case rates from negative binomial regression models at the census block group level in urban Miami-Dade County, across the five study periods. Color shows the direction of each association: blue for fewer reported cases and red for more reported cases. Shading shows the significance level (*** p < 0.001, ** p < 0.01, * p < 0.05, (.) p < 0.10). A cell is blank when a variable did not enter the final model for that period. Full estimates, including incidence rate ratios, 95% confidence intervals, and model diagnostics, appear in Supporting Information S4 Table.

Several demographic and household characteristics were consistently associated with lower reported COVID-19 case rates across CBGs. CBGs with higher proportions of children demonstrated persistent protective effects throughout all study periods. Specifically, CBGs with more children under age 5 exhibited significantly lower case rates, with incidence rate ratios (IRRs) ranging from 0.986 to 0.989 (*p* < 0.01), while those with more children aged 5–17 showed IRRs between 0.992 and 0.993 (*p* < 0.01). CBGs with higher percentages of Black or African American residents were also associated with lower case rates from Period 3 onwards (IRR: 0.991–0.995, *p* < 0.001). A similar protective association was observed for those identifying as ‘Some other race’ during Periods 1–4, with IRRs between 0.993 and 0.996 (p-values ranging from 0.005 to 0.07). Additional protective effects were identified in areas with greater proportions of renter-occupied units during Periods 2–4 (IRR: 0.998–0.999, *p* < 0.05), and in CBGs with higher percentages of ‘other family’ households during Periods 2–5 (IRR: 0.996, *p* < 0.01).

Several associations were specific to particular phases of the pandemic. In early periods (Periods 1−2), higher infection rates were linked to lower educational attainment (no high school diploma: IRR: 1.004–1.005, *p* < 0.01), and commuting alone by car, truck, or van (IRR: 1.003–1.004, *p* < 0.01). Conversely, Hispanic ethnicity (Period 1, IRR: 0.997, *p* = 0.03) and foreign-born status (Periods 1−2, IRR: 0.995–0.998, *p* < 0.01 to *p* = 0.06) were associated with lower rates. In later periods, having a high school diploma (Periods 3−4, IRR: 0.997, *p* = 0.06 to *p* = 0.08) and Medicaid enrollment (Periods 3 & 5, IRR: 0.996–0.997, p = 0.02 to p = 0.05) emerged as protective.

Linguistic factors showed complex associations. CBGs with greater proportions of residents speaking other Indo-European languages showed lower infection rates in Periods 2, 4, and 5 (IRR: 0.995–0.996, *p* < 0.01 to *p* = 0.04), and Spanish-speaking CBGs exhibited lower rates in later periods (IRR: 0.996, *p* < 0.01), but Spanish speaking households with limited English proficiency were marginally associated with higher infection rates (IRR: 1.002–1.021, *p* = 0.07 to *p* = 0.1). Household overcrowding, measured as the percentage of housing units with more than two occupants per room, entered the candidate set for every model but was retained in none.

### Socio-demographic profiles of COVID-19 hot spots and cold spots

#### Spatial cluster identification.

Spatial analysis using local *G*_i_* statistics identified statistically significant geographic clusters of elevated (hot spots) and suppressed (cold spots) COVID-19 case rates across the five defined study periods (Fig 3). The number of CBGs identified as hot spots ranged from 83 (4.5% of total) in Period 5 to a peak of 121 (6.6%) in Period 3. In contrast, cold spots consistently encompassed a larger spatial extent, increasing from 134 CBGs (7.3%) in Period 1 to 273 (14.9%) in Period 2. Cold spots covered 7.6% to 15.0% of the county population and hot spots 3.6% to 5.7%, highlighting uneven patterns of geographic vulnerability and protection over time. Table 4 compares the socio-demographic characteristics of identified clusters to the countywide profile. Statistically significant differences, determined via Mann-Whitney U tests, indicate distinct social and structural conditions associated with sustained hot and cold spot designations.

**Table 4:** Descriptive statistics of COVID-19 hot and cold spots. Values highlighted in bold with significance levels in parentheses indicate whether a cluster’s characteristic is different from that of the entire county, assessed by Mann-Whitney U-tests.

	Period 1		Period 2		Period 3		Period 4		Period 5		
CBG Characteristic	Hot Spot	Cold Spot	Hot Spot	Cold Spot	Hot Spot	Cold Spot	Hot Spot	Cold Spot	Hot Spot	Cold Spot	Miami-Dade County
Number of CBGs [% of total]	96 [5.2]	134 [7.3]	90 [4.9]	273 [14.9]	121 [6.6]	253 [13.8]	98 [5.4]	209 [11.4]	83 [4.5]	146 [8.0]	1,831 [100]
Population [% of total]	120,706 [4.7]	194,922 [7.6]	103,248 [4.0]	362,390 [14.1]	146,914 [5.7]	386,128 [15.0]	122,638 [4.8]	336,045 [13.1]	92,552 [3.6]	244,814 [9.5]	2,570,609
*Median Pct Hispanic*	69	62 (*)	71	56 (***)	68	42.5 (***)	69.5	43 (***)	68	61.5 (***)	73
*Median Pct Black*	2	1 (***)	2	2 (**)	1 (*)	42.5 (***)	1	30 (***)	1 (**)	7 (***)	3
*Median Pct Below Poverty*	11	9 (***)	13	10 (**)	9 (*)	16 (***)	11	14 (**)	9	13	12
*Median Pct No HS*	19 (.)	8 (***)	19 (.)	7 (***)	9 (**)	19 (***)	13	17 (*)	13 (.)	12.5	14
*Median MedHHIncome [US$]*	55,676 (.)	73,780 (***)	52,022 (*)	69,663 (***)	77,069 (***)	48,383 (***)	74,643 (*)	52,056 (***)	69,417	56,136	60,364
*Median Pct No Health Insurance*	14.5	10 (**)	12.5	11 (**)	12 (.)	19 (***)	12	18 (***)	12 (.)	17 (**)	13
*Median Pct Limited English*	22	16	24.5 (*)	16 (**)	15 (*)	19	18	21	17	24	19
*Median Pct Renter*	60 (***)	38 (.)	52 (*)	47	48 (*)	57 (***)	40.5	57 (***)	47	55 (*)	42
*Median Pct under 18*	18.7	19.9	18.5	19.8 (.)	18.9	22.9 (***)	20.8	21.8 (**)	19.3	20.8 (.)	19.4
*Median Pct 65 or above*	18.5	16.5	19.0 (.)	16.9	13.1 (*)	13.1 (***)	13.9 (.)	14.3 (***)	17.6	14.5 (**)	16.7

(***): p < 0.001 (Highly Significant); (**): 0.001 ≤ p < 0.01 (Very Significant); (*): 0.01 ≤ p < 0.05 (Significant); (.): 0.05 ≤ p < 0.1 (Marginally significant).

**Fig 3:**
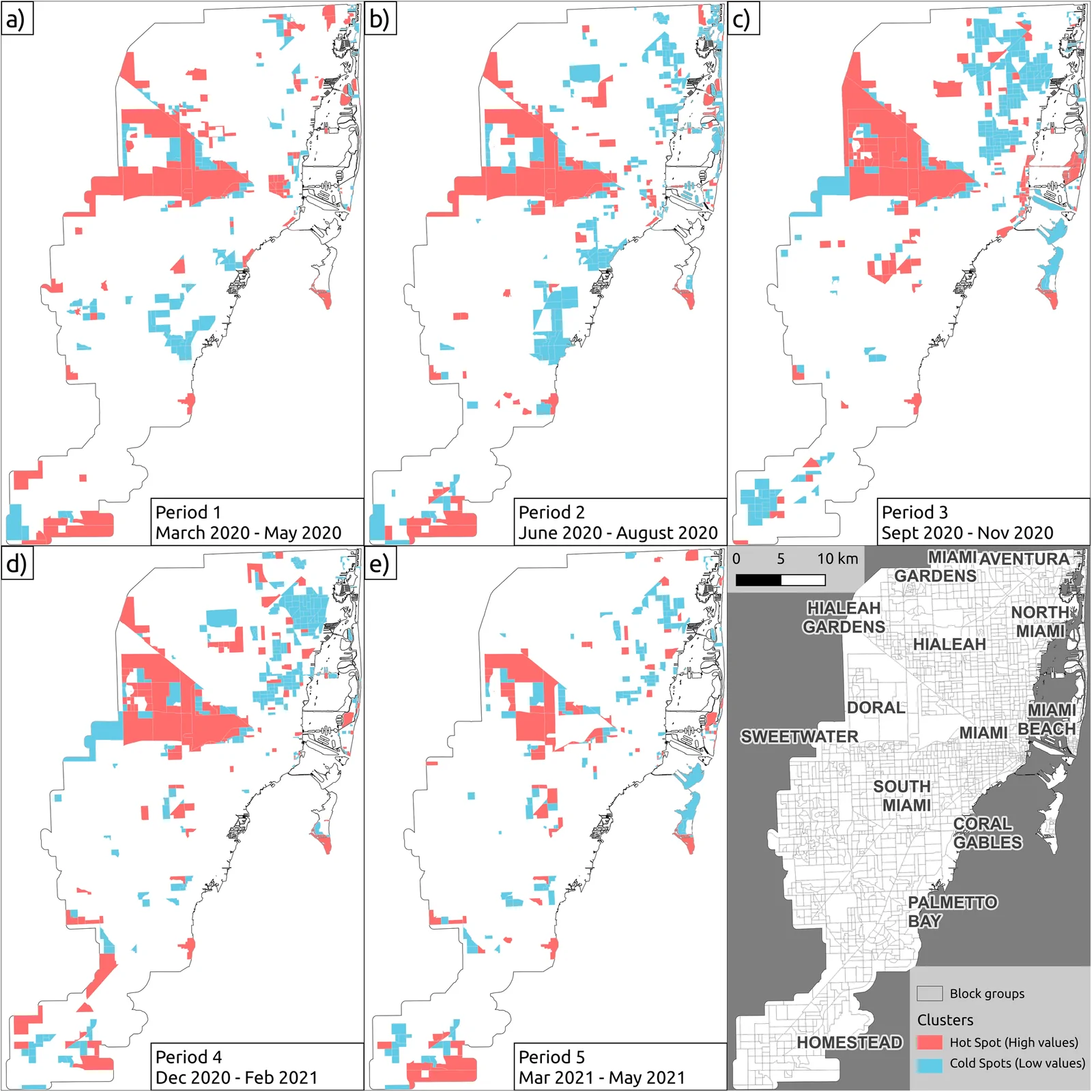
COVID-19 hot spots (red, high-value clusters) and cold spots (blue, low-value clusters) in urban Miami-Dade County in five study periods (a-e). Block group boundaries and water features are from the U.S. Census Bureau TIGER/Line files (public domain).

#### Temporal evolution of cluster characteristics.

The composition of spatial clusters evolved significantly over time, reflecting shifts in the geography of census block group-level risk (see Table 4). In the early stages of the pandemic (Periods 1–2), hot spots emerged in more vulnerable CBGs with lower median household incomes ($55,676 in Period 1, *p* = 0.06; $52,022 in Period 2, *p* = 0.03) and higher renter-occupancy (60% in Period 1, *p* < 0.001; 52% in Period 2, *p* = 0.02). In contrast, early cold spots were concentrated in CBGs with significantly higher incomes ($73,780 and $69,663 in Periods 1 and 2, respectively; both *p* < 0.001).

Starting in Period 3, a striking reversal in spatial patterns became evident. Hot spots shifted to higher-income CBGs ($77,069 in Period 3, *p* < 0.001), while cold spots became concentrated in historically Black CBGs along the I-95 corridor, including Liberty City, Little Haiti, West Little River and parts of the municipalities of Opa-Locka, Miami Gardens, North Miami and North Miami Beach (Fig 4). These cold spots were characterized by significantly higher proportions of Black residents (median: 42.5% vs. 3% countywide, *p* < 0.001), lower median incomes ($48,383, *p* < 0.001) and higher uninsured residents (19% vs. 13%, *p* < 0.001), a pattern that persisted through Period 5.

**Fig 4:**
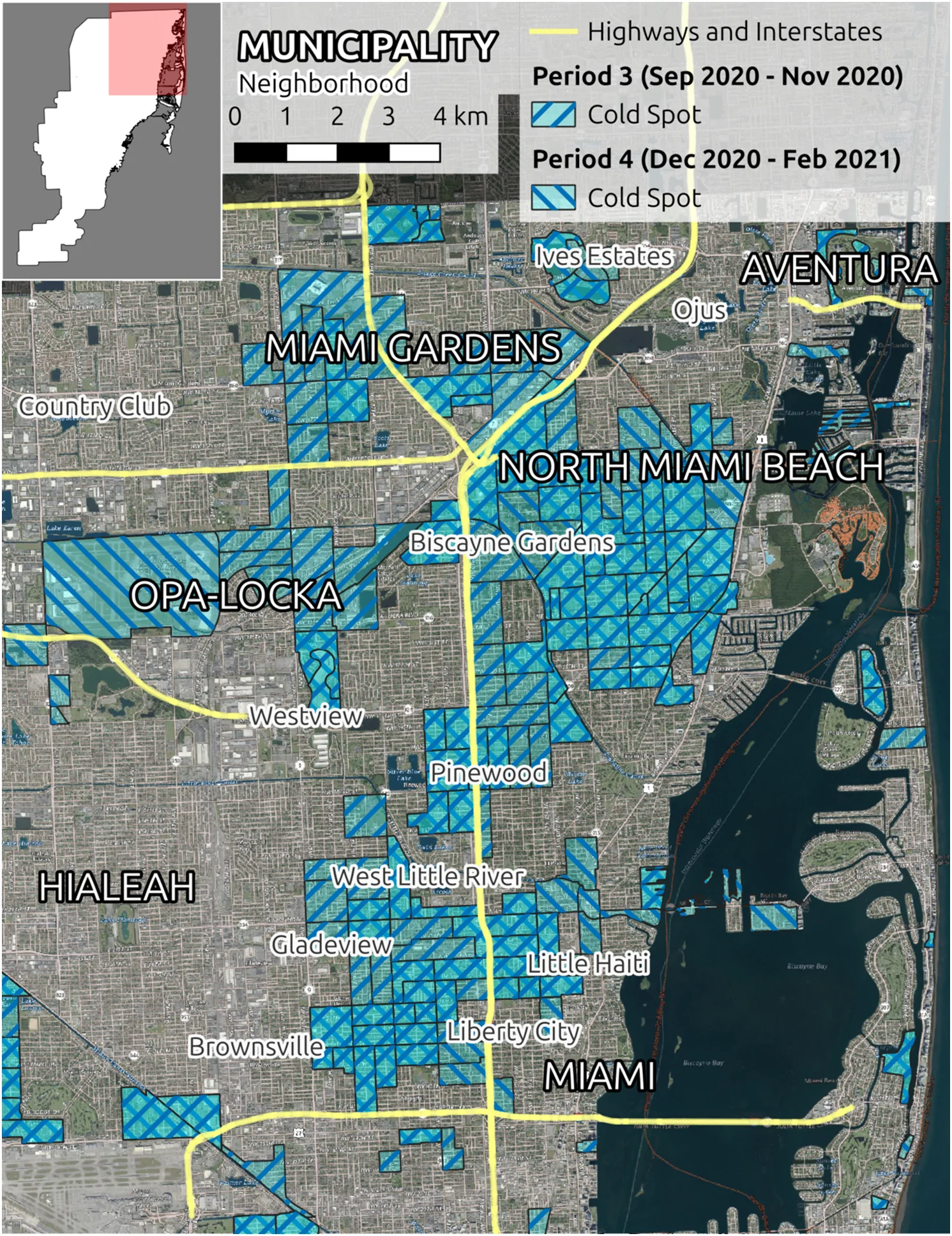
Locations of cold spots in Northeast Miami-Dade County that emerged in Periods 3 and 4 (September 2020 to February 2021), predominantly in neighborhoods with a higher proportion of low-income Black or African American residents. Background imagery courtesy of U.S. Geological Survey. Block group boundaries and roads are from the U.S. Census Bureau TIGER/Line files (public domain).

### Socio-demographic predictors of COVID-19 hot spot and cold spot membership

To examine the predictors of spatial cluster membership, logistic regression models were estimated separately for each study period and cluster type (hot spot and cold spot). All models incorporated spatial eigenvector filtering to account for spatial dependence. Statistically significant predictors are presented in Fig 5, with full model specifications provided in Supporting Information S5 Table. In early study periods, household composition and socioeconomic status were the primary predictors of cluster assignment. In Period 1, CBGs with higher proportions of married couple families (odds ratio [OR]: 1.03, *p* < 0.001), single-person households (OR: 1.03, *p* < 0.001) and renter-occupied housing (OR: 1.02, *p* < 0.001) were more likely to be classified as hot spots. Conversely, higher median household income (per US$10,000 increment) significantly predicted cold spot membership in Period 1 (OR: 1.10, *p* < 0.001) and Period 2 (OR: 1.08, *p* < 0.001), while lower educational attainment (i.e., greater proportions of residents without a high school diploma) was inversely associated with cold spot status (OR: 0.96–0.98, *p* < 0.01) during those periods. Recoding the 20 top-coded block groups to US$250,000 and refitting on 1,628 block groups left the direction and significance of the income association intact. The odds ratio for cold spot membership was 1.09 in Period 1 (*p* < 0.001) and 1.05 in Period 2, where significance weakened from *p* < 0.001 to *p* < 0.01.

**Fig 5:**
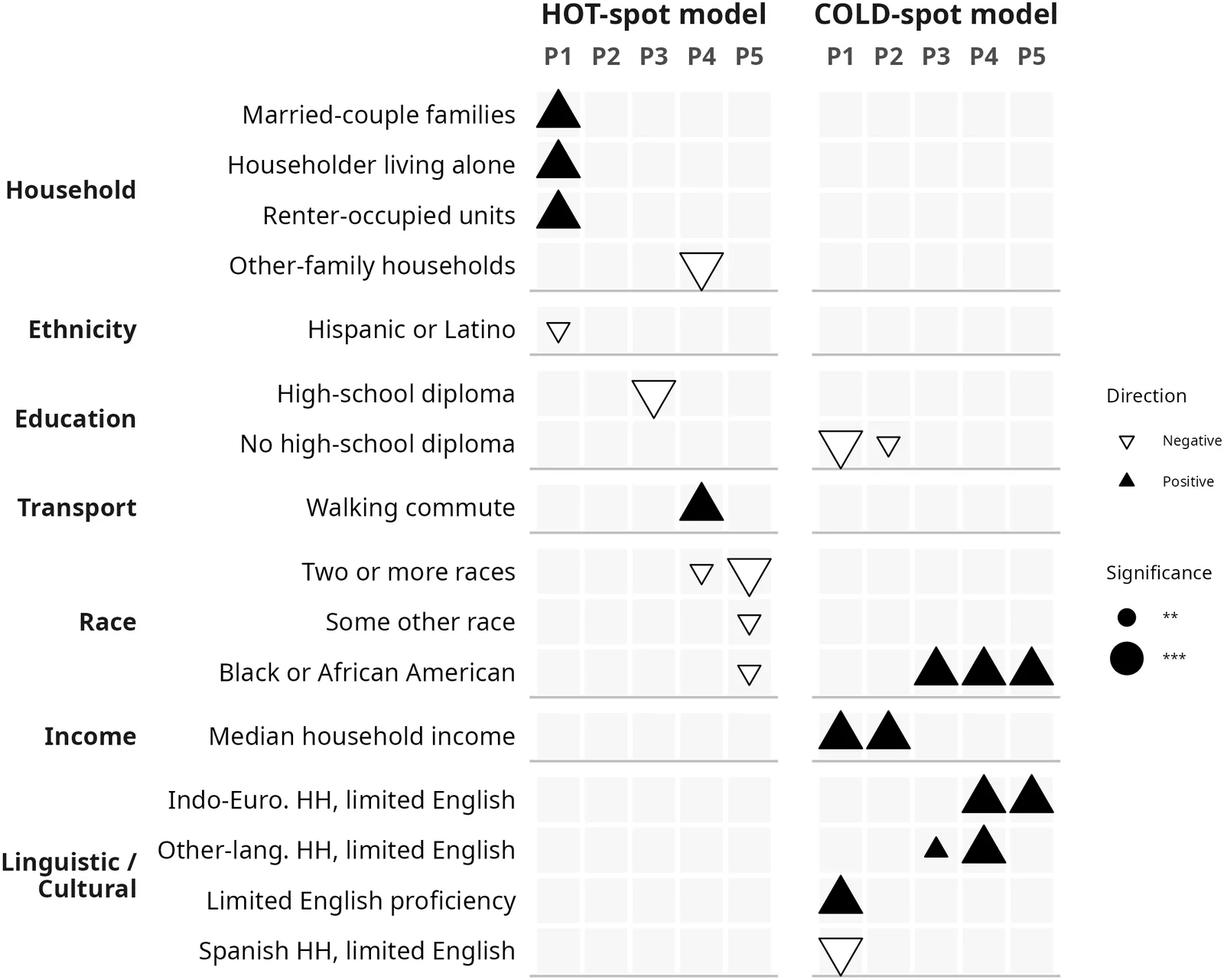
Predictors of hot and cold spot cluster membership by study period. Two-panel plot of block group-level logistic regression models. The left panel represents the hot spot model; the right panel represents the cold spot model. Upward pointing triangles indicate factors associated with increased odds of hot or cold spot membership. Downward triangles indicate factors associated with decreased odds of hot or cold spot membership. Size denotes significance levels (*** p < 0.001, ** p < 0.01). Periods range from P1 (March-May 2020) to P5 (March-May 2021).

In later periods, racial composition emerged as the most salient predictor. From Period 3 through Period 5, higher percentages of Black residents were consistently and strongly associated with cold spot membership (OR: 1.01–1.04, *p* < 0.001). By Period 5, this variable also significantly reduced the odds of hot spot classification (OR: 0.98, *p* < 0.01), marking a spatial bifurcation in pandemic exposure and reporting. In these later periods, linguistic and racialized access factors also gained importance. CBGs with greater proportions of households speaking non-English languages and reporting limited English proficiency were increasingly likely to be classified as cold spots.

## Discussion

This study advances understanding of how the COVID-19 pandemic unfolded across diverse urban neighborhoods by examining fine-grained spatiotemporal dynamics in Miami-Dade County, Florida. By using census block group (CBG)-level data, we identified notable shifts in reported case patterns and in the demographic and socioeconomic determinants of high (hot spot) and low (cold spot) infection rates across five distinct phases of the pandemic. Our findings reveal that reported case rates, spatial clustering, and their underlying predictors evolved markedly over time, shaped not only by transmission dynamics but also by infrastructure availability, demographic composition, and healthcare-seeking behaviors.

### The testing access paradox: Geographic patterns in disease detection

Our analysis revealed a striking temporal shift in COVID-19 detection patterns across Miami-Dade County. While initial hot spots concentrated in lower-income areas, these gradually transitioned to higher-income neighborhoods as testing expanded. Simultaneously, historically Black neighborhoods along the I-95 corridor emerged as persistent cold spots despite multiple indicators of elevated disease risk, such as lower median income, reduced insurance coverage, and limited educational attainment.

This geographic inversion aligns with findings from multiple metropolitan areas where expanded testing capacity failed to translate into proportional utilization [21,60]. Census block groups with documented risk factors, including higher renter occupancy, larger Black populations, and greater Medicaid enrollment, showed protective associations with reported rates as the pandemic progressed. This epidemiological paradox, documented across diverse settings [61,62], suggests that passive surveillance systems capture healthcare utilization patterns rather than true disease distribution. Holden et al. [63] estimated 50–80% of infections went undetected, with gaps likely concentrated in communities facing access barriers; patterns our fine-scale analysis confirms at the neighborhood level.

Read together, the cold spots and the affluent hot spots describe one gradient of access rather than two separate effects. Where testing was hard to reach, true infections in the corridor neighborhoods went uncounted, and where testing was easy to reach and easy to justify, reported counts in more affluent areas were lifted by testing pursued for reasons only loosely tied to illness, including return-to-work clearance, travel, and contact follow-up. Serosurveys that compare antibody prevalence with reported cases show both ends of this gradient, with lower detection in lower-income areas and more frequent, largely non-clinical testing among higher-income groups even when symptom-driven testing is similar across them [64], so our reported-case surface over-ascertains higher-income areas and under-ascertains lower-income ones at the same time. Both distortions sit on top of real transmission, since the affluent experienced genuine waves and the early low-income hot spots were largely real, so the gradient shapes the reported geography without erasing it.

### Factors influencing test-seeking behavior across communities

The emergence of cold spots in high-risk neighborhoods reflects complex interactions between infrastructure, community characteristics, and test-seeking behaviors. Despite expanded testing capacity, accessing sites required time, transportation, and flexibility that varied considerably across neighborhoods. The I-95 corridor communities, with lower incomes, higher uninsured rates, and public transit dependence, faced compounded barriers through appointment systems, administrative processes, and work schedule conflicts that disproportionately affected essential workers.

The counterintuitive relationship between risk factors and reported rates indicates systematic detection bias. Areas with higher proportions of renters, Black residents, and Medicaid enrollees showed lower reported rates, and block groups with limited English proficiency were more likely to fall in cold spots. This improbability suggests that our surveillance system captured healthcare utilization differences rather than disease distribution. Similar patterns in other cities confirm this phenomenon [61,65], sometimes described as the inverse care law, in which those who need care the least tend to get the most, and those who need care the most, tend to get the least [66].

The association between “Other Indo-European languages” and lower reported rates reflects Miami-Dade's linguistic complexity. This category (7.2% of county population) encompasses Haitian Creole speakers (~4.9%), Portuguese-speaking Brazilians, and European language speakers, communities with divergent healthcare access patterns [67]. This aggregation may obscure important differences in testing utilization, highlighting limitations of available census categories.

### Methodological advances and implications for spatial epidemiology

The shift from ZIP code to census block group analysis proved essential for detecting spatial paradoxes invisible at broader scales. This granular approach revealed neighborhood-level disparities within areas often assumed homogeneous, demonstrating how aggregation masks important variations in disease detection.

Spatial eigenvector filtering (SEF) allowed us to isolate true demographic associations from spatial artifacts by decomposing autocorrelation into orthogonal components [58,59]. This proved particularly valuable given Miami-Dade's complex geography, where neighboring areas share infrastructure that could confound traditional analyses.

These innovations have broader implications for surveillance research. The temporal instability of risk associations, with identical characteristics predicting opposite outcomes across phases, reveals fundamental limitations of static vulnerability indices. Future spatial epidemiology should incorporate dynamic measures of healthcare access alongside conventional risk factors, prioritizing fine-scale analyses that distinguish true disease patterns from detection artifacts.

### Rethinking pandemic preparedness: From passive to active surveillance

Our findings necessitate fundamental surveillance accommodations. The systematic detection gaps in Miami-Dade demonstrate that expanding testing without addressing utilization barriers can worsen disparities. Future preparedness must shift from passive availability to active approaches to ensure comprehensive coverage.

Effective surveillance requires complementary detection streams independent of voluntary test-seeking: wastewater monitoring for population-level tracking [68–70], syndromic surveillance capturing severe cases [71,72], and random sampling [73,74] providing unbiased prevalence estimates. Geographic deployment should prioritize accessibility, incorporating transit routes, work schedules, and utilization patterns, in addition to population density. Mobile units should target areas with high vulnerability but low reported rates and minimize barriers related to inflexible schedules.

Real-time monitoring can flag neighborhoods where testing falls below risk-adjusted thresholds, enabling rapid intervention. The I-95 corridor's transformation into cold spots exemplifies how static vulnerability indices miss evolving patterns. Building comprehensive surveillance requires viewing detection disparities as central challenges requiring intentional solutions, not unfortunate side effects.

### Limitations and future directions

Our analysis faces several constraints. We examined reported cases without corresponding testing rate data, preventing direct quantification of detection gaps. Testing site locations were not separable in the surveillance records, so the geography of testing capacity could not be modeled directly. This constraint is itself a feature of passive surveillance, which records where cases were reported rather than where testing was available. While geocoding successfully captured self-reported residential addresses, three-month periods may obscure weekly variations. The 2017–2021 ACS estimates predate pandemic conditions, missing relevant population shifts.

The five study periods were defined to align with documented shifts in testing capacity and public health policy, but the design is descriptive and does not isolate the effect of any single intervention. The changes in reported cases across periods reflect the combined influence of transmission dynamics, population behavior, testing availability, variant emergence, and policy, and these contributions cannot be separated within three-month observational windows. Behavior also changes voluntarily as case counts rise, independent of mandates, and no established method cleanly attributes outcomes to multiple interventions introduced at the same time [75]. We therefore read the periods as a temporal frame for describing spatial patterns of detection, not as an evaluation of policy.

Methodological limitations include sensitivity to geographic boundary definitions [76,77] and the inability to infer individual behaviors from area-level data. Census categories aggregating diverse communities, notably “Other Indo-European languages” combining Haitian Creole and European speakers, mask important heterogeneity. Workplace exposures, mobility patterns, and individual behaviors remain unmeasured confounders. The transportation variables warrant similar caution. They capture commute mode among workers, not vehicle access across the population, and without testing-rate data we cannot tell whether their associations with reported cases, such as the early tie between car commuting and higher rates, reflect exposure or access to testing, an ambiguity consistent with the detection paradox. The overcrowding measure calls for the same care. It entered no final model in any period. That absence does not show that crowding failed to shape transmission, only that it added nothing to models already containing related housing and language measures.

These constraints highlight research priorities: seroprevalence studies quantifying undercounting, testing site accessibility modeling, and mixed-methods approaches addressing behavioral factors. Future surveillance could employ machine learning to estimate true burden from biased detection data, while wastewater monitoring offers testing-independent prevalence estimates. However, technical solutions alone cannot overcome access-rooted disparities. Building surveillance systems that perform well across communities remains essential for effective pandemic response.

These findings come from a single county over fifteen months, and the specific geography may not transfer to other settings. The mechanism behind the detection paradox, a surveillance signal that follows access to testing rather than the burden of disease, does not depend on local features, so similar detection gradients are likely wherever testing is distributed unevenly and for other conditions tracked through passive surveillance.

## Conclusions

This spatiotemporal analysis of 1,831 Miami-Dade County census block groups reveals how pandemic surveillance can systematically misrepresent disease distribution. The detection paradox, hot spots shifting from low-income to affluent areas while historically Black neighborhoods became cold spots, suggests that reported case geography reflected healthcare utilization patterns rather than transmission dynamics.

Traditional risk factors paradoxically predicted lower reported rates as testing expanded, signaling measurement failure when renter occupancy, Black population share, and Medicaid enrollment appeared protective. Fine-scale analysis with spatial eigenvector filtering exposed patterns invisible at coarser scales, while temporal instability of associations revealed limitations of static vulnerability indices.

These findings suggest reconceptualizing surveillance from passive observation to active design. Future systems must integrate multiple detection streams operating independently of voluntary test-seeking, prioritize accessibility in geographic deployment, and monitor for risk-adjusted detection gaps. Miami-Dade's experience demonstrates that comprehensive surveillance requires intentional design to illuminate rather than obscure disease patterns; a fundamental requirement for effective pandemic response.

Glossary:

- Cold Spot: A statistically significant spatial cluster of low values (i.e., low COVID-19 infection rates) identified through the local *G*_i_* statistic.
- Empirical Bayes (EB) Smoothing: A statistical technique used to stabilize rates in areas with small populations by adjusting them toward a local mean.
- Hot Spot: A statistically significant spatial cluster of high values (i.e., high COVID-19 infection rates) identified through the local *G*_i_* statistic.
- Spatial Eigenvector Filtering (SEF): A method that uses eigenvectors from a spatial weights matrix to control for spatial autocorrelation in regression models.
- Urban Development Boundary (UDB): The official Miami-Dade County line separating areas designated for urban development from rural and environmental lands.

## Supporting information

S1 Fig Study area, Miami-Dade County. (a) Spatial extent of the study region and population density within the Urban Development Boundary that defines the block groups included in the analysis. (b) Spatial distribution of reported cases. Block group boundaries, roads, and water features are from the U.S. Census Bureau TIGER/Line files (public domain). The Florida counties inset also uses TIGER/Line boundaries, and the detailed shoreline is from the Miami-Dade County Open Data Hub.
(PDF)

S2 Fig First 15 spatial eigenvector filters (SEF_1 to SEF_15) for the study area. These maps display orthogonal components of spatial structure, ordered from the broadest scale (SEF_1, a constant baseline) to increasingly finer scales of geographic variation. Block group boundaries are from the U.S. Census Bureau TIGER/Line files (public domain).
(PDF)

S1 Table Policy context. Timeline of relevant events, orders, and policies by study period.
(DOCX)

S2 Table Social determinants of health variables assessed at the census block group level. (DOCX)

S3 Table Summary statistics of socio-economic variables in census block groups within urban Miami-Dade County. (DOCX)

S4 Table Final negative binomial regression models for Periods 1–5 (March 2020 to May 2021). Significance codes: *** p < 0.001, ** p < 0.01, * p < 0.05,. p < 0.1.
(DOCX)

S5 Table Final logistic regression models for Periods 1–5 (March 2020 to May 2021). Significance codes: *** p < 0.001, ** p < 0.01, * p < 0.05,. p < 0.1.
(DOCX)

S1 File Supplementary Methods. Model specifications and variable definitions for the Getis-Ord Gi* statistic, the negative binomial regression, and the logistic regression models.
(DOCX)

S1 Checklist STROBE checklist for cross-sectional studies, completed for an ecological design. (DOCX)
