# Supplementary material for: The detection paradox: How COVID-19 surveillance patterns shifted from lower-income to more affluent neighborhoods in Miami-Dade County
Source: PLoS One. 2026 Oct 23;21(10):e0361211. doi: 10.1371/journal.pone.0361211 (PMC13693879; doi:10.1371/journal.pone.0361211)
Supplement: Supplementary file 1 [file pone.0361211.s001.pdf]

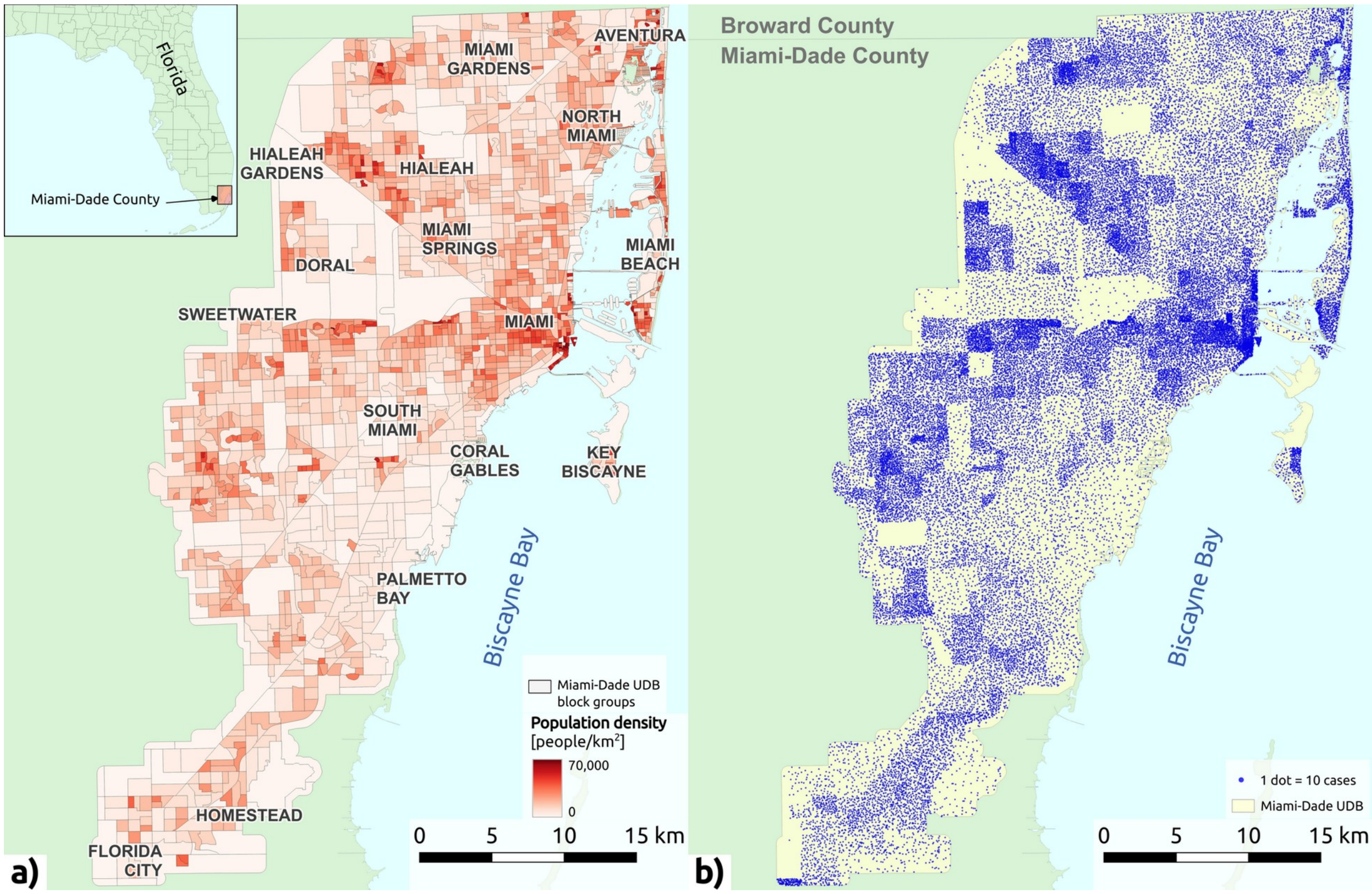

Florida
Miami-Dade County
MIAMI GARDENS
AVENTURA
NORTH MIAMI
HIALEAH GARDENS
HIALEAH
MIAMI SPRINGS
DORAL
MIAMI BEACH
SWEETWATER
MIAMI
SOUTH MIAMI
CORAL GABLES
KEY BISCAYNE
PALMETTO BAY
Biscayne Bay
Miami-Dade UDB block groups
Population density [people/km²]
70,000
0
HOMESTEAD
FLORIDA CITY
0 5 10 15 km
a)
Broward County
Miami-Dade County
Biscayne Bay
1 dot = 10 cases
Miami-Dade UDB
0 5 10 15 km
b)
