# Supplementary material for: The detection paradox: How COVID-19 surveillance patterns shifted from lower-income to more affluent neighborhoods in Miami-Dade County
Source: PLoS One. 2026 Oct 23;21(10):e0361211. doi: 10.1371/journal.pone.0361211 (PMC13693879; doi:10.1371/journal.pone.0361211)
Supplement: Supplementary file 2 [file pone.0361211.s002.pdf]

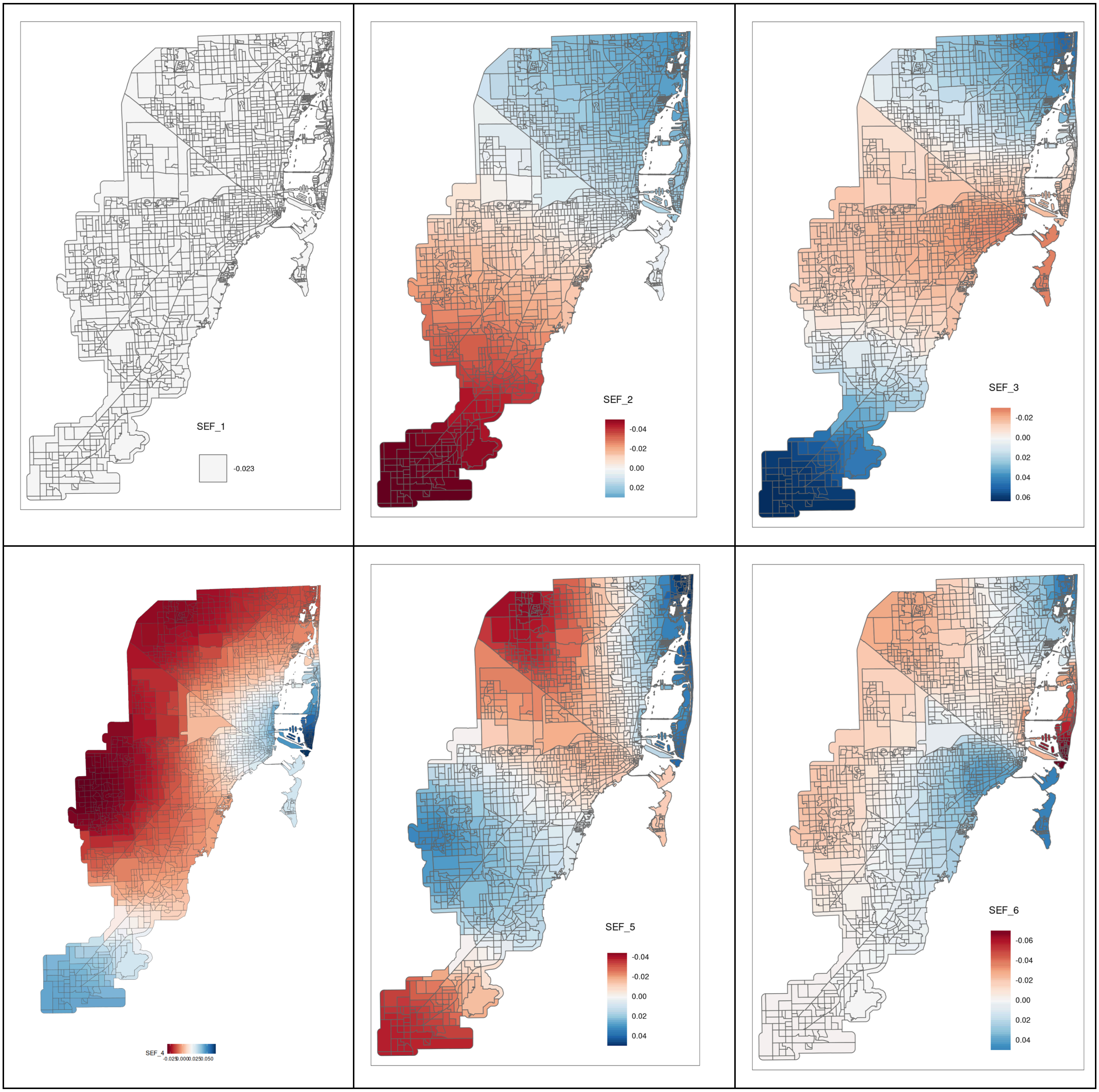

SEF_1
-0.023
SEF_2
-0.04
-0.02
0.00
0.02
SEF_3
-0.02
0.00
0.02
0.04
0.06
SEF_4
-0.025
0.000
0.025
0.050
SEF_5
-0.04
-0.02
0.00
0.02
0.04
SEF_6
-0.06
-0.04
-0.02
0.00
0.02
0.04

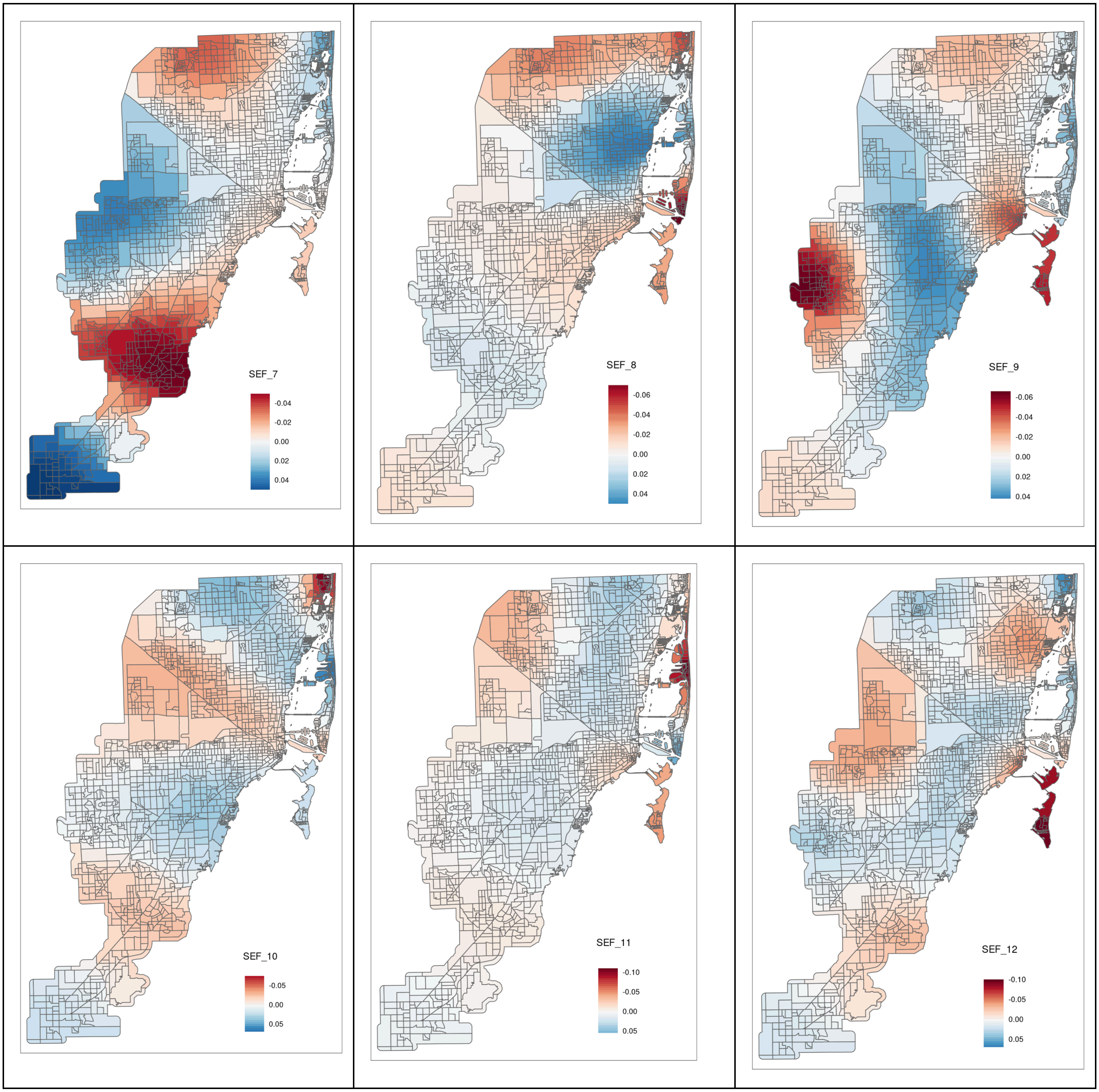
SEF_7
-0.04
-0.02
0.00
0.02
0.04
SEF_8
-0.06
-0.04
-0.02
0.00
0.02
0.04
SEF_9
-0.06
-0.04
-0.02
0.00
0.02
0.04
SEF_10
-0.05
0.00
0.05
SEF_11
-0.10
-0.05
0.00
0.05
SEF_12
-0.10
-0.05
0.00
0.05

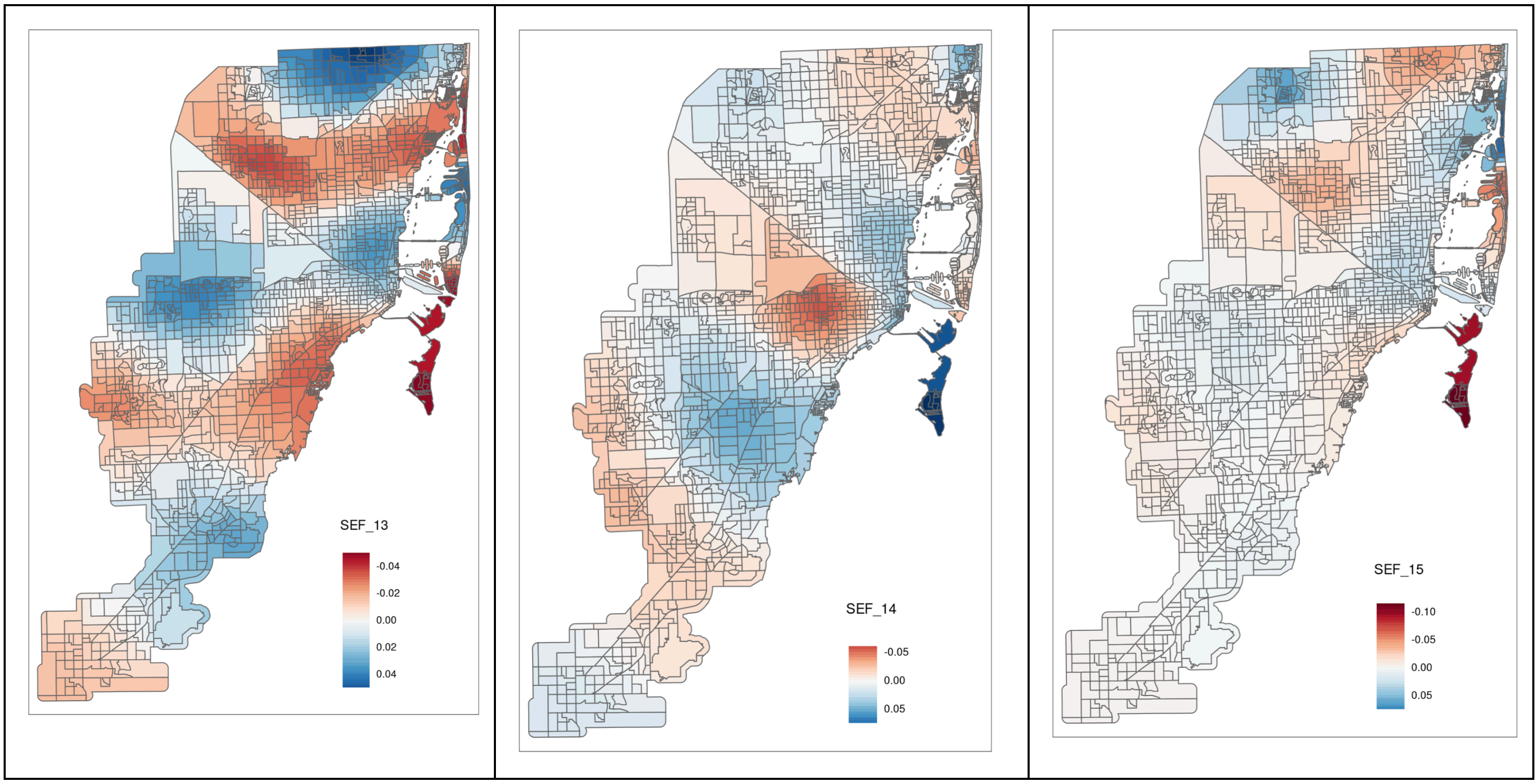
SEF_13
-0.04
-0.02
0.00
0.02
0.04
SEF_14
-0.05
0.00
0.05
SEF_15
-0.10
-0.05
0.00
0.05
