# Supplementary material for: The detection paradox: How COVID-19 surveillance patterns shifted from lower-income to more affluent neighborhoods in Miami-Dade County
Source: PLoS One. 2026 Oct 23;21(10):e0361211. doi: 10.1371/journal.pone.0361211 (PMC13693879; doi:10.1371/journal.pone.0361211)
Supplement: Supplementary file 3 [file pone.0361211.s003.docx]

**S1 Table**. **Policy context.** Timeline of relevant events, orders, and policies by study period.

| **Date** | **Jurisdiction** | **Event** |
| --- | --- | --- |
| Mar 12, 2020 | Miami-Dade County | State of Emergency declared |
| March 2020 | Miami-Dade County | First confirmed COVID-19 cases and deaths reported in the county |
| Mar 19, 2020 | Miami-Dade County | Shutdown of nonessential services, businesses, parks, and facilities |
| Mar 26, 2020 | Miami-Dade County | “Safer at Home” order reinforced |
| Mar 27, 2020 | Miami-Dade County | First drive-thru testing site opens (initially for 65+ with symptoms) |
| Apr 1, 2020 | Florida | Statewide stay-at-home order issued by the Governor |
| Early April 2020 | Miami-Dade County | Testing expands to healthcare workers and first responders |
| Apr 9, 2020 | Miami-Dade County | Indoor mask mandate begins |
| Late Apr–Early May 2020 | Miami-Dade County | Closures of adult daycares |
| May 2020 | Miami-Dade County | “New Normal” color-coded reopening plan announced |
| May 15, 2020 | Miami-Dade County | Phased reopening begins |
| May 27, 2020 | Miami-Dade County | Outdoor dining allowed |
| June 2020 | Miami-Dade County | More facilities reopen with approved safety plans; beaches reopen |
| Jul 2, 2020 | Miami-Dade County | Curfew and beach closures reinstated |
| July 2020 | Miami-Dade County | Indoor dining and entertainment venues closed again due to case surge |
| Summer 2020 | Miami-Dade County | First major COVID-19 wave (non-variant), peaked in July |
| Aug–Sep 2020 | Miami-Dade County & Florida | Most businesses reopen; Florida enters Phase 3 of reopening |
| Sep 26, 2020 | Florida | Phase 3 reopening order issued statewide |
| Late 2020 | Miami-Dade County | Schools begin staggered return to in-person learning |
| Dec 14, 2020 | Florida | First vaccine doses administered (Pfizer; for healthcare workers) |
| Dec 23, 2020 | Florida | Vaccines extended to long-term care facility residents and staff |
| Jan 6, 2021 | Miami-Dade County | Phase 3 guidelines for services in Miami Beach published |
| January 2021 | Florida | Vaccines become available to people aged 65+ |
| Mar 1, 2021 | Florida | Vaccines open to teachers, law enforcement, and firefighters 50+ |
| Mar 15, 2021 | Florida | Vaccines become available for those aged 60+ |
| Mar 29, 2021 | Florida | Vaccines become available for those aged 40+ |
| Apr 5, 2021 | Florida | Vaccines open to all adults (18+) |
| Apr 6, 2021 | Miami-Dade County | Order 33-20 restores some restrictions |
| April–May 2021 | Miami-Dade County | State of Emergency extended weekly |
| May 3, 2021 | Florida | Governor suspends all local COVID-19 restrictions, effective July 1 |
| May 12, 2021 | Nationwide (FDA) | Pfizer vaccine approved for ages 12–15 |
