# Supplementary material for: The detection paradox: How COVID-19 surveillance patterns shifted from lower-income to more affluent neighborhoods in Miami-Dade County
Source: PLoS One. 2026 Oct 23;21(10):e0361211. doi: 10.1371/journal.pone.0361211 (PMC13693879; doi:10.1371/journal.pone.0361211)
Supplement: Supplementary file 4 [file pone.0361211.s004.docx]

**S2 Table.** Social determinants of health variables assessed at the census block group level.

| SDOH Measured | Description | Reference* |
| --- | --- | --- |
| Race | *Pct. of Asian Population* | % of White population |
|  | *Pct. of Native Hawaiian and Other Pacific Islander Population* |  |
|  | *Pct. of Black or African American Population* |  |
|  | *Pct. of Population Identifying as Some Other Race* |  |
|  | *Pct. of Population Identifying as Two or More Races* |  |
| Age | *Pct. of Population Under 5 Years Old* | % of Population 18 - 64 |
|  | *Pct. of Population Aged 5 to 17 Years* |  |
|  | *Pct. of Population Aged 65 Years or Over* |  |
| Education | *Pct. of Population with No High School Diploma (Age 25+)* | % of population with undergraduate or associate degrees |
|  | *Pct. of Population with High School Diploma or Equivalent (Age 25+)* |  |
|  | *Pct. of Population with Some College or Associate's Degree (Age 25+)* |  |
|  | *Pct. of Population with a Graduate or Professional Degree (Age 25+)* |  |
| Ethnicity | *Pct. of Hispanic or Latino Population* | - |
| Income | *Pct. of Population Below Poverty Level* | - |
|  | *Median Household Income* |  |
| Housing | *Pct. of Renter-Occupied Housing Units* | - |
|  | *Pct. of Housing Units with >2 Occupants Per Room (Overcrowding)* |  |
| Healthcare Access | *Pct. of Population with No Health Insurance Coverage* | - |
|  | *Pct. of Population Enrolled in Medicaid* |  |
| Empl. Status | *Pct. of Workers Who Worked From Home* | - |
| Transportation | *Pct. of Workers Commuting by Car, Truck, or Van (Alone)* | - |
|  | *Pct. of Workers Commuting by Carpool* |  |
|  | *Pct. of Workers Commuting by Public Transportation* |  |
|  | *Pct. of Workers Commuting by Bicycle* |  |
|  | *Pct. of Workers Commuting by Walking* |  |
| Household Structure | *Pct. of Family Households that are Married-Couple Families* | - |
|  | *Pct. of Family Households that are Other Family Types* |  |
|  | *Pct. of Family Households: Female Householder, No Spouse Present* |  |
|  | *Pct. of Nonfamily Households: Householder Living Alone* |  |
| Linguistic, Cultural Geographic Origin | *Pct. of Foreign-Born Population* | - |
|  | *Pct. of Population with Limited English Proficiency* |  |
|  | *Pct. of Population Speaking Spanish* |  |
|  | *Pct. of Households: Spanish-Speaking with Limited English* |  |
|  | *Pct. of Population Speaking Other Indo-European Languages* |  |
|  | *Pct. of Households: Other Indo-European Speaking with Limited English* |  |
|  | *Pct. of Households: Other Language Speaking with Limited English* |  |
