# Supplementary material for: The detection paradox: How COVID-19 surveillance patterns shifted from lower-income to more affluent neighborhoods in Miami-Dade County
Source: PLoS One. 2026 Oct 23;21(10):e0361211. doi: 10.1371/journal.pone.0361211 (PMC13693879; doi:10.1371/journal.pone.0361211)
Supplement: Supplementary file 5 [file pone.0361211.s005.docx]

**S3 Table.** Summary statistics of socio-economic variables in census block groups within urban Miami-Dade County

| **Category** | **Variable** | **Mean** | **Median** | **Min.** | **Max.** |
| --- | --- | --- | --- | --- | --- |
| Race | *Pct Asian* | 1.69 | 0 | 0 | 49 |
|  | *Pct Native Hawaiian and Other Pacific Islander* | 0.02 | 0 | 0 | 9 |
|  | *Pct Black or African American* | 15.5 | 3 | 0 | 100 |
|  | *Pct Some Other Race* | 5.18 | 3 | 0 | 51 |
|  | *Pct Two or More Races* | 19.1 | 17 | 0 | 70 |
| Ethnicity | *Pct Hispanic* | 66.4 | 73 | 0 | 100 |
| Age | *Pct Under 5* | 5.4 | 5 | 0 | 34 |
|  | *Pct 5 to 17* | 14.0 | 13 | 0 | 62 |
|  | *Pct 65 or Above* | 17.5 | 16 | 0 | 100 |
| Education | *Pct No High School* | 16.5 | 14 | 0 | 88 |
|  | *Pct High School or Equivalent* | 25.4 | 25 | 0 | 72 |
|  | *Pct Some College* | 14.8 | 13.5 | 0 | 68 |
|  | *Pct Graduate degree* | 13.4 | 9 | 0 | 91 |
| Income | *Pct Below Poverty* | 15.5 | 12 | 0 | 79 |
|  | *Median HH Income [US $]* | 70,354 | 60,364 | 7,487 | 249,706 |
| Housing characteristics | *Pct Renter* | 44.7 | 42 | 0 | 100 |
|  | *Pct 2 or More Occ. Per Room* | 0.6 | 0 | 0 | 44 |
| Healthcare Access | *Pct No Health Insurance* | 14.9 | 13 | 0 | 78 |
|  | *Pct Medicaid* | 19.5 | 18 | 0 | 78 |
| Employment | *Pct Worked from Home* | 9.1 | 6 | 0 | 100 |
| Transportation | *Pct Car, Truck, Van Alone* | 82.2 | 86 | 0 | 100 |
|  | *Pct Car, Truck, Van, Carpooled* | 8.7 | 7 | 0 | 100 |
|  | *Pct Public Transportation* | 3.8 | 0 | - | 57 |
|  | *Pct Bicycle* | 0.6 | 0 | 0 | 55 |
|  | *Pct Walked* | 2.1 | 0 | 0 | 55 |
| Household | *Pct Family Households, Married Couple* | 45 | 44 | 0 | 100 |
| Structure | *Pct Family Households, Other family types* | 24.9 | 24 | 0 | 100 |
|  | *Pct Family Households, Female Householder, No spouse* | 17.6 | 16 | 0 | 100 |
|  | *Pct Nonfamily households, Alone* | 24.3 | 21 | 0 | 100 |
| Linguistic / | *Pct Foreign Born* | 53.11 | 55 | 0 | 98 |
| Cultural / | *Pct Limited English* | 22.4 | 19 | 0 | 94 |
| Geo origin | *Pct Spanish, limited English* | 10.7 | 16 | 0 | 94 |
|  | *Pct Other Indo European* | 7.7 | 3 | 0 | 100 |
|  | *Pct Other Indo-European, Limited English* | 1.4 | 0 | 0 | 60 |
|  | *Pct Other Language, Limited english* | 0.1 | 0 | 0 | 21 |
