# Supplementary material for: The detection paradox: How COVID-19 surveillance patterns shifted from lower-income to more affluent neighborhoods in Miami-Dade County
Source: PLoS One. 2026 Oct 23;21(10):e0361211. doi: 10.1371/journal.pone.0361211 (PMC13693879; doi:10.1371/journal.pone.0361211)
Supplement: Supplementary file 6 [file pone.0361211.s006.docx]

# S4 Table. Final negative binomial regression models for Periods 1 to 5 (March 2020 to May 2021). Significance codes: *** p < 0.001, ** p < 0.01, * p < 0.05, . p < 0.1.

**S4 Table, Period 1.** Final Negative Binomial Regression Model for Period 1 (March 2020 - May 2020). Variables with SEF_ prefix represent spatial eigenvectors to account for spatial dependence

| **Variable** | **Coeff.** | **Std. Err.** | **IRR** | **95% CI** | **p-value** | Signif. level |
| --- | --- | --- | --- | --- | --- | --- |
| (Intercept) | -4.296 | 0.163 | 0.014 | [0.01, 0.019] | 4.82E-153 | *** |
| pct_native_hawaiian_and_other_pacific_islander | -0.084 | 0.054 | 0.920 | [0.828, 1.022] | 1.20E-01 |  |
| pct_black_or_african_american | -0.003 | 0.001 | 0.997 | [0.995, 1] | 6.78E-02 | . |
| pct_some_other_race | -0.007 | 0.002 | 0.993 | [0.988, 0.998] | 4.93E-03 | ** |
| pct_hispanic_or_latino | -0.003 | 0.002 | 0.997 | [0.994, 1] | 3.21E-02 | * |
| pct_under_5 | -0.011 | 0.004 | 0.989 | [0.982, 0.996] | 2.43E-03 | ** |
| pct_5_to_17 | -0.008 | 0.002 | 0.992 | [0.987, 0.997] | 1.16E-03 | ** |
| pct_no_high_school | 0.005 | 0.002 | 1.005 | [1.001, 1.008] | 5.61E-03 | ** |
| pct_car_truck_van | 0.004 | 0.001 | 1.004 | [1.001, 1.007] | 3.88E-03 | ** |
| pct_bicycle | 0.018 | 0.008 | 1.018 | [1.002, 1.033] | 2.34E-02 | * |
| pct_family_households_other_family_female_householder_no_spouse | -0.005 | 0.002 | 0.995 | [0.992, 0.998] | 1.72E-03 | ** |
| pct_foreign_born | -0.005 | 0.002 | 0.995 | [0.992, 0.998] | 2.53E-03 | ** |
| pct_limited_english | -0.017 | 0.011 | 0.983 | [0.961, 1.005] | 1.37E-01 |  |
| pct_spanish_limited_english_household | 0.021 | 0.011 | 1.021 | [0.998, 1.044] | 6.91E-02 | . |
| pct_other_indo_european_limited_english_household | 0.022 | 0.012 | 1.022 | [0.998, 1.047] | 7.16E-02 | . |
| SEF_2 | 2.347 | 0.853 | 10.450 | [1.964, 55.598] | 5.93E-03 | ** |
| SEF_4 | 1.509 | 0.926 | 4.522 | [0.736, 27.77] | 1.03E-01 |  |
| SEF_5 | -4.809 | 0.906 | 0.008 | [0.001, 0.048] | 1.13E-07 | *** |
| SEF_8 | 3.351 | 0.815 | 28.536 | [5.781, 140.856] | 3.89E-05 | *** |
| SEF_10 | -1.734 | 0.762 | 0.177 | [0.04, 0.787] | 2.30E-02 | * |
| SEF_11 | 2.655 | 0.855 | 14.219 | [2.659, 76.041] | 1.91E-03 | ** |
| SEF_13 | 2.050 | 0.740 | 7.768 | [1.821, 33.14] | 5.61E-03 | ** |
| SEF_14 | -1.430 | 0.756 | 0.239 | [0.054, 1.052] | 5.85E-02 | . |

| **Model diagnostics:** | AIC | 11266 |
| --- | --- | --- |
|  | theta | 2.961 |
|  | Std. error | 0.126 |
|  | Residual deviance | 1713.9 |
|  | df | 1585 |
|  | 2 x log likelihood | -11218.371 |
| Without spatial filtering | Moran's I statistic | 0.11 |
|  | p-value | 0 |
| With spatial filtering | Moran's I statistic | 0.088 |
|  | p-value | 0 |

**S4 Table, Period 2.** Final Negative Binomial Regression Model for Period 2 (June 2020 - August 2020). Variables with SEF_ prefix represent spatial eigenvectors to account for spatial dependence

| **Variable** | **Coeff.** | **Std. Err.** | **IRR** | **95% CI** | **p-value** | Signif. level |
| --- | --- | --- | --- | --- | --- | --- |
| (Intercept) | -2.826 | 0.120 | 0.059 | [0.047, 0.075] | 8.98E-122 | *** |
| pct_native_hawaiian_and_other_pacific_islander | -0.079 | 0.046 | 0.924 | [0.844, 1.011] | 8.49E-02 | . |
| pct_some_other_race | -0.004 | 0.002 | 0.996 | [0.992, 1] | 6.30E-02 | . |
| pct_under_5 | -0.014 | 0.003 | 0.986 | [0.98, 0.993] | 2.64E-05 | *** |
| pct_5_to_17 | -0.007 | 0.002 | 0.993 | [0.989, 0.997] | 7.49E-04 | *** |
| pct_no_high_school | 0.004 | 0.001 | 1.004 | [1.001, 1.007] | 3.71E-03 | ** |
| pct_renter | -0.001 | 0.001 | 0.999 | [0.998, 1] | 4.47E-02 | * |
| pct_car_truck_van | 0.003 | 0.001 | 1.003 | [1.001, 1.006] | 4.59E-03 | ** |
| pct_family_households_other_family | -0.004 | 0.001 | 0.996 | [0.994, 0.999] | 1.22E-03 | ** |
| pct_foreign_born | -0.002 | 0.001 | 0.998 | [0.995, 1] | 6.42E-02 | . |
| pct_spanish_limited_english_household | 0.002 | 0.001 | 1.002 | [1, 1.005] | 9.52E-02 | . |
| pct_other_indo_european | -0.005 | 0.001 | 0.995 | [0.992, 0.998] | 8.41E-04 | *** |
| SEF_2 | -2.316 | 0.700 | 0.099 | [0.025, 0.389] | 9.45E-04 | *** |
| SEF_5 | -8.351 | 0.739 | 0.000 | [0, 0.001] | 1.37E-29 | *** |
| SEF_6 | -1.460 | 0.682 | 0.232 | [0.061, 0.883] | 3.21E-02 | * |
| SEF_7 | 1.503 | 0.630 | 4.494 | [1.308, 15.437] | 1.70E-02 | * |
| SEF_8 | 3.365 | 0.685 | 28.945 | [7.554, 110.917] | 9.10E-07 | *** |
| SEF_9 | -1.776 | 0.635 | 0.169 | [0.049, 0.588] | 5.18E-03 | ** |
| SEF_10 | -2.806 | 0.671 | 0.060 | [0.016, 0.225] | 2.91E-05 | *** |
| SEF_11 | 2.187 | 0.716 | 8.905 | [2.19, 36.213] | 2.25E-03 | ** |
| SEF_13 | 2.490 | 0.642 | 12.064 | [3.426, 42.475] | 1.06E-04 | *** |
| SEF_14 | -2.473 | 0.663 | 0.084 | [0.023, 0.309] | 1.90E-04 | *** |
| SEF_15 | 1.418 | 0.659 | 4.128 | [1.134, 15.025] | 3.15E-02 | * |

| **Model diagnostics:** | AIC | 16211 |
| --- | --- | --- |
|  | theta | 3.188 |
|  | Std. error | 0.115 |
|  | Residual deviance | 1716.1 |
|  | df | 1585 |
|  | 2 x log likelihood | -16162.763 |
| Without spatial filtering | Moran's I statistic | 0.042 |
|  | p-value | 0.002 |
| With spatial filtering | Moran's I statistic | 0.015 |
|  | p-value | 0.15 |

**S4 Table, Period 3.** Final Negative Binomial Regression Model for Period 3 (September 2020 - November 2020). Variables with SEF_ prefix represent spatial eigenvectors to account for spatial dependence

| **Variable** | **Coeff.** | **Std. Err.** | **IRR** | **95% CI** | **p-value** | Signif. level |
| --- | --- | --- | --- | --- | --- | --- |
| (Intercept) | -2.450 | 0.134 | 0.086 | [0.066, 0.112] | 5.43E-75 | *** |
| pct_black_or_african_american | -0.009 | 0.001 | 0.991 | [0.989, 0.993] | 6.92E-13 | *** |
| pct_some_other_race | -0.004 | 0.002 | 0.996 | [0.992, 1] | 7.14E-02 | . |
| pct_two_plus_races | -0.002 | 0.001 | 0.998 | [0.995, 1] | 7.07E-02 | . |
| pct_under_5 | -0.014 | 0.004 | 0.986 | [0.98, 0.993] | 9.87E-05 | *** |
| pct_5_to_17 | -0.007 | 0.002 | 0.993 | [0.989, 0.998] | 5.85E-03 | ** |
| pct_65_or_over | -0.004 | 0.002 | 0.996 | [0.993, 1] | 6.04E-02 | . |
| pct_high_school_eq | -0.003 | 0.001 | 0.997 | [0.995, 1] | 7.71E-02 | . |
| pct_some_college | -0.003 | 0.002 | 0.997 | [0.994, 1.001] | 1.70E-01 |  |
| pct_renter | -0.002 | 0.001 | 0.998 | [0.997, 1] | 2.21E-02 | * |
| pct_no_health_insurance | -0.003 | 0.002 | 0.997 | [0.994, 1.001] | 1.32E-01 |  |
| pct_medicaid | -0.004 | 0.002 | 0.996 | [0.993, 0.999] | 1.70E-02 | * |
| pct_family_households_other_family | -0.004 | 0.001 | 0.996 | [0.993, 0.998] | 7.09E-04 | *** |
| pct_nonfamily_households_householder_alone | 0.002 | 0.001 | 1.002 | [1, 1.005] | 8.25E-02 | . |
| pct_foreign_born | -0.002 | 0.001 | 0.998 | [0.995, 1.001] | 1.59E-01 |  |
| pct_limited_english | -0.006 | 0.004 | 0.994 | [0.987, 1.002] | 1.27E-01 |  |
| pct_spanish | -0.004 | 0.001 | 0.996 | [0.994, 0.999] | 8.90E-03 | ** |
| pct_spanish_limited_english_household | 0.006 | 0.004 | 1.006 | [0.999, 1.013] | 1.15E-01 |  |
| SEF_3 | -2.365 | 0.776 | 0.094 | [0.021, 0.43] | 2.30E-03 | ** |
| SEF_4 | -1.924 | 0.784 | 0.146 | [0.031, 0.679] | 1.42E-02 | * |
| SEF_5 | -3.680 | 0.727 | 0.025 | [0.006, 0.105] | 4.17E-07 | *** |
| SEF_6 | -2.375 | 0.677 | 0.093 | [0.025, 0.351] | 4.55E-04 | *** |
| SEF_7 | 1.646 | 0.642 | 5.188 | [1.473, 18.268] | 1.04E-02 | * |
| SEF_10 | -2.725 | 0.665 | 0.066 | [0.018, 0.241] | 4.12E-05 | *** |
| SEF_12 | -1.167 | 0.654 | 0.311 | [0.086, 1.122] | 7.44E-02 | . |
| SEF_13 | 2.701 | 0.635 | 14.895 | [4.288, 51.747] | 2.13E-05 | *** |

| **Model diagnostics:** | AIC | 14099 |
| --- | --- | --- |
|  | theta | 3.448 |
|  | Std. error | 0.132 |
|  | Residual deviance | 1710.9 |
|  | df | 1582 |
|  | 2 x log likelihood | -14044.58 |
| Without spatial filtering | Moran's I statistic | 0.055 |
|  | p-value | 0 |
| With spatial filtering | Moran's I statistic | 0.023 |
|  | p-value | 0.06 |

**S4 Table, Period 4.** Final Negative Binomial Regression Model for Period 4 (December 2020 - February 2021). Variables with SEF_ prefix represent spatial eigenvectors to account for spatial dependence

| **Variable** | **Coeff.** | **Std. Err.** | **IRR** | **95% CI** | **p-value** | Signif. level |
| --- | --- | --- | --- | --- | --- | --- |
| (Intercept) | -1.779 | 0.109 | 0.169 | [0.136, 0.209] | 2.23E-60 | *** |
| pct_black_or_african_american | -0.006 | 0.001 | 0.994 | [0.992, 0.996] | 3.90E-07 | *** |
| pct_some_other_race | -0.005 | 0.002 | 0.995 | [0.991, 1] | 3.15E-02 | * |
| pct_two_plus_races | -0.002 | 0.001 | 0.998 | [0.996, 1.001] | 1.66E-01 |  |
| pct_under_5 | -0.014 | 0.003 | 0.986 | [0.98, 0.992] | 5.37E-06 | *** |
| pct_5_to_17 | -0.008 | 0.002 | 0.992 | [0.988, 0.996] | 1.90E-04 | *** |
| pct_65_or_over | -0.003 | 0.002 | 0.997 | [0.994, 1.001] | 1.01E-01 |  |
| pct_high_school_eq | -0.003 | 0.001 | 0.997 | [0.995, 1] | 5.59E-02 | . |
| pct_renter | -0.002 | 0.001 | 0.998 | [0.997, 0.999] | 2.14E-03 | ** |
| pct_no_health_insurance | -0.002 | 0.002 | 0.998 | [0.994, 1.001] | 1.31E-01 |  |
| pct_family_households_other_family | -0.004 | 0.001 | 0.996 | [0.994, 0.998] | 3.95E-04 | *** |
| pct_limited_english | -0.015 | 0.009 | 0.985 | [0.967, 1.003] | 1.02E-01 |  |
| pct_spanish | -0.004 | 0.001 | 0.996 | [0.994, 0.999] | 2.41E-03 | ** |
| pct_spanish_limited_english_household | 0.016 | 0.009 | 1.016 | [0.998, 1.036] | 8.36E-02 | . |
| pct_other_indo_european | -0.004 | 0.002 | 0.996 | [0.993, 1] | 4.17E-02 | * |
| pct_other_indo_european_limited_english_household | 0.016 | 0.010 | 1.016 | [0.996, 1.036] | 1.26E-01 |  |
| SEF_2 | -1.261 | 0.689 | 0.283 | [0.073, 1.094] | 6.73E-02 | . |
| SEF_4 | -1.579 | 0.745 | 0.206 | [0.048, 0.887] | 3.40E-02 | * |
| SEF_5 | -4.291 | 0.720 | 0.014 | [0.003, 0.056] | 2.52E-09 | *** |
| SEF_6 | -3.306 | 0.658 | 0.037 | [0.01, 0.133] | 4.95E-07 | *** |
| SEF_7 | 1.807 | 0.619 | 6.090 | [1.808, 20.507] | 3.54E-03 | ** |
| SEF_10 | -3.039 | 0.637 | 0.048 | [0.014, 0.167] | 1.82E-06 | *** |
| SEF_13 | 1.534 | 0.622 | 4.635 | [1.368, 15.7] | 1.37E-02 | * |
| SEF_14 | -1.348 | 0.636 | 0.260 | [0.075, 0.903] | 3.40E-02 | * |

| **Model diagnostics:** | AIC | 16081 |
| --- | --- | --- |
|  | theta | 3.445 |
|  | Std. error | 0.123 |
|  | Residual deviance | 1703 |
|  | df | 1584 |
|  | 2 x log likelihood | -16750.916 |
| Without spatial filtering | Moran's I statistic | 0.03 |
|  | p-value | 0.022 |
| With spatial filtering | Moran's I statistic | 0.005 |
|  | p-value | 0.35 |

**S4 Table, Period 5.** Final Negative Binomial Regression Model for Period 5 (March 2021 - May 2021). Variables with SEF_ prefix represent spatial eigenvectors to account for spatial dependence

| **Variable** | **Coeff.** | **Std. Err.** | **IRR** | **95% CI** | **p-value** | Signif. level |
| --- | --- | --- | --- | --- | --- | --- |
| (Intercept) | -2.505 | 0.098 | 0.082 | [0.067, 0.099] | 6.74E-145 | *** |
| pct_black_or_african_american | -0.005 | 0.001 | 0.995 | [0.993, 0.998] | 6.83E-05 | *** |
| pct_under_5 | -0.011 | 0.003 | 0.989 | [0.983, 0.995] | 7.43E-04 | *** |
| pct_5_to_17 | -0.007 | 0.002 | 0.993 | [0.989, 0.997] | 1.05E-03 | ** |
| pct_renter | -0.001 | 0.001 | 0.999 | [0.998, 1] | 7.79E-02 | . |
| pct_medicaid | -0.003 | 0.002 | 0.997 | [0.994, 1] | 4.48E-02 | * |
| pct_family_households_other_family | -0.004 | 0.001 | 0.996 | [0.994, 0.999] | 1.47E-03 | ** |
| pct_foreign_born | -0.002 | 0.001 | 0.998 | [0.995, 1.001] | 1.26E-01 |  |
| pct_limited_english | -0.014 | 0.010 | 0.986 | [0.967, 1.004] | 1.32E-01 |  |
| pct_spanish | -0.004 | 0.001 | 0.996 | [0.994, 0.999] | 5.53E-03 | ** |
| pct_spanish_limited_english_household | 0.015 | 0.010 | 1.015 | [0.996, 1.035] | 1.12E-01 |  |
| pct_other_indo_european | -0.004 | 0.002 | 0.996 | [0.992, 1] | 3.81E-02 | * |
| pct_other_indo_european_limited_english_household | 0.016 | 0.010 | 1.017 | [0.996, 1.037] | 1.12E-01 |  |
| pct_high_school_eq | -0.002 | 0.001 | 0.998 | [0.995, 1.001] | 1.39E-01 |  |
| SEF_3 | 1.306 | 0.732 | 3.692 | [0.879, 15.503] | 7.43E-02 | . |
| SEF_5 | -2.812 | 0.714 | 0.060 | [0.015, 0.243] | 8.14E-05 | *** |
| SEF_6 | -2.291 | 0.636 | 0.101 | [0.029, 0.352] | 3.18E-04 | *** |
| SEF_7 | 2.087 | 0.626 | 8.059 | [2.365, 27.463] | 8.50E-04 | *** |
| SEF_10 | -1.986 | 0.658 | 0.137 | [0.038, 0.498] | 2.53E-03 | ** |
| SEF_12 | -1.568 | 0.638 | 0.208 | [0.06, 0.728] | 1.40E-02 | * |
| SEF_13 | 2.016 | 0.629 | 7.508 | [2.189, 25.751] | 1.35E-03 | ** |
| SEF_14 | -0.955 | 0.645 | 0.385 | [0.109, 1.363] | 1.39E-01 |  |

| **Model diagnostics:** | AIC | 14770 |
| --- | --- | --- |
|  | theta | 3.496 |
|  | Std. error | 0.131 |
|  | Residual deviance | 1711 |
|  | df | 1586 |
|  | 2 x log likelihood | -14723.809 |
| Without spatial filtering | Moran's I statistic | 0.029 |
|  | p-value | 0.028 |
| With spatial filtering | Moran's I statistic | 0.009 |
|  | p-value | 0.254 |
