# Supplementary material for: The detection paradox: How COVID-19 surveillance patterns shifted from lower-income to more affluent neighborhoods in Miami-Dade County
Source: PLoS One. 2026 Oct 23;21(10):e0361211. doi: 10.1371/journal.pone.0361211 (PMC13693879; doi:10.1371/journal.pone.0361211)
Supplement: Supplementary file 7 [file pone.0361211.s007.docx]

# S5 Table. Final logistic regression models for Periods 1 to 5 (March 2020 to May 2021). Significance codes: *** p < 0.001, ** p < 0.01, * p < 0.05, . p < 0.1.

**S5 Table, Period 1.** Final Logistic Regression Models for Period 1 (March 2020 - May 2020). Variables with SEF_ prefix represent spatial eigenvectors to account for spatial dependence

| **Period 1: March 2020 - May 2020** | | | | | | | | |
| --- | --- | --- | --- | --- | --- | --- | --- | --- |
|  |  |  |  |  |  |  |  |  |
| **Logistic regression results predicting if a block group belongs to a hot spot** | | | | | | | | |
| **Variable** | **Estimate** | **Std. Error** | **z value** | **Pr(>\|z\|)** | **Signif.** | **Odds Ratio (OR)** | **95% CI (Lower)** | **95% CI (Upper)** |
| **(Intercept)** | -5.261 | 0.746 | -7.051 | 1.78E-12 | *** | 0.01 | 0 | 0.02 |
| **pct_hispanic_or_latino** | -0.015 | 0.005 | -2.926 | 3.43E-03 | ** | 0.99 | 0.98 | 1 |
| **pct_renter** | 0.018 | 0.005 | 3.634 | 2.79E-04 | *** | 1.02 | 1.01 | 1.03 |
| **pct_family_households_married_couple_family** | 0.032 | 0.009 | 3.422 | 6.21E-04 | *** | 1.03 | 1.01 | 1.05 |
| **pct_nonfamily_households_householder_alone** | 0.032 | 0.009 | 3.656 | 2.56E-04 | *** | 1.03 | 1.01 | 1.05 |
| **SEF_5** | -28.386 | 6.208 | -4.572 | 4.82E-06 | *** | 0 | 0 | 0 |
| **SEF_10** | -18.647 | 5.273 | -3.536 | 4.06E-04 | *** | 0 | 0 | 0 |
|  |  |  |  |  |  |  |  |  |
| **Logistic regression results predicting if a block group belongs to a cold spot** | | | | | | | | |
| **Variable** | Estimate | Std. Error | z value | Pr(>\|z\|) | Signif. | Odds Ratio (OR) | 95% CI (Lower) | 95% CI (Upper) |
| **(Intercept)** | -3.256 | 0.374 | -8.708 | 3.08E-18 | *** | 0.04 | 0.02 | 0.08 |
| **pct_no_high_school** | -0.044 | 0.012 | -3.754 | 1.74E-04 | *** | 0.96 | 0.93 | 0.98 |
| **pct_limited_english** | 0.08 | 0.016 | 4.919 | 8.72E-07 | *** | 1.08 | 1.05 | 1.12 |
| **pct_spanish_limited_english_household** | -0.063 | 0.015 | -4.127 | 3.68E-05 | *** | 0.94 | 0.91 | 0.97 |
| **SEF_10** | -13 | 3.771 | -3.449 | 5.64E-04 | *** | 0 | 0 | 0 |
| **median_household_income (per $10,000)** | 0.095 | 0.027 | 3.545 | 3.92E-04 | *** | 1.099 | 1.043 | 1.159 |

**S5 Table, Period 2.** Final Logistic Regression Models for Period 2 (June 2020 - August 2020). Variables with SEF_ prefix represent spatial eigenvectors to account for spatial dependence

| **Period 2: June 2020 - August 2020** | | | | | | | | |
| --- | --- | --- | --- | --- | --- | --- | --- | --- |
|  |  |  |  |  |  |  |  |  |
| **Logistic regression results predicting if a block group belongs to a hot spot** | | | | | | | | |
| **Variable** | **Estimate** | **Std. Error** | **z value** | **Pr(>\|z\|)** | **Signif.** | **Odds Ratio (OR)** | **95% CI (Lower)** | **95% CI (Upper)** |
| **(Intercept)** | -3.332 | 0.156 | -21.308 | < 2E-16 | *** | 0.04 | 0.03 | 0.05 |
| **SEF_4** | 17.151 | 5.207 | 3.294 | 9.88E-04 | *** | 2.81E+07 | 1038.36 | 7.61E+11 |
| **SEF_5** | -23.767 | 5.57 | -4.267 | 1.98E-05 | *** | 0 | 0 | 0 |
| **SEF_10** | -22.875 | 6 | -3.813 | 1.37E-04 | *** | 0 | 0 | 0 |
|  |  |  |  |  |  |  |  |  |
| **Logistic regression results predicting if a block group belongs to a cold spot** | | | | | | | | |
| **Variable** | **Estimate** | **Std. Error** | **z value** | **Pr(>\|z\|)** | **Signif.** | **Odds Ratio (OR)** | **95% CI (Lower)** | **95% CI (Upper)** |
| **(Intercept)** | -2.339 | 0.253 | -9.23 | < 2E-16 | *** | 0.1 | 0.06 | 0.16 |
| **pct_no_high_school** | -0.024 | 0.008 | -3.05 | 2.29E-03 | ** | 0.98 | 0.96 | 0.99 |
| **median_household_income (per $10,000)** | 0.075 | 0.020 | 3.773 | 1.61E-04 | *** | 1.077 | 1.037 | 1.12 |
| **SEF_2** | 25 | 3.647 | 6.854 | 7.20E-12 | *** | 7.20E+10 | 5.66E+07 | 9.14E+13 |
| **SEF_3** | 11.83 | 3.172 | 3.731 | 1.91E-04 | *** | 1.37E+05 | 273.94 | 68830000 |
| **SEF_4** | 27.34 | 4.096 | 6.675 | 2.48E-11 | *** | 7.50E+11 | 2.44E+08 | 2.30E+15 |
| **SEF_6** | 22.42 | 3.344 | 6.707 | 1.99E-11 | *** | 5.46E+09 | 7.77E+06 | 3.84E+12 |
| **SEF_9** | 16.88 | 4.022 | 4.197 | 2.70E-05 | *** | 2.15E+07 | 8159.09 | 5.63E+10 |

**S5 Table, Period 3.** Final Logistic Regression Models for Period 3 (September 2020 - November 2020). Variables with SEF_ prefix represent spatial eigenvectors to account for spatial dependence

| **Period 3: September 2020 - November 2020** | | | | | | | | |
| --- | --- | --- | --- | --- | --- | --- | --- | --- |
|  |  |  |  |  |  |  |  |  |
| **Logistic regression results predicting if a block group belongs to a hot spot** | | | | | | | | |
| **Variable** | **Estimate** | **Std. Error** | **z value** | **Pr(>\|z\|)** | **Signif.** | **Odds Ratio (OR)** | **95% CI (Lower)** | **95% CI (Upper)** |
| **(Intercept)** | -2.09 | 0.266 | -7.856 | 3.98E-15 | *** | 0.12 | 0.07 | 0.21 |
| **pct_high_school_eq** | -0.047 | 0.01 | -4.956 | 7.20E-07 | *** | 0.95 | 0.94 | 0.97 |
| **SEF_2** | 19.871 | 6.099 | 3.258 | 1.12E-03 | ** | 4.27E+08 | 2742.07 | 6.63E+13 |
| **SEF_4** | 23.86 | 6.262 | 3.811 | 1.39E-04 | *** | 2.30E+10 | 1.08E+05 | 4.90E+15 |
| **SEF_5** | -34.828 | 7.333 | -4.749 | 2.04E-06 | *** | 0 | 0 | 0 |
| **SEF_9** | 22.518 | 6.195 | 3.635 | 2.78E-04 | *** | 5.99E+09 | 32074.6 | 1.03E+15 |
| **SEF_12** | -22.743 | 4.71 | -4.828 | 1.38E-06 | *** | 0 | 0 | 0 |
| **SEF_13** | 22.781 | 4.706 | 4.841 | 1.29E-06 | *** | 7.83E+09 | 7.73E+05 | 2.92E+13 |
|  |  |  |  |  |  |  |  |  |
| **Logistic regression results predicting if a block group belongs to a cold spot** | | | | | | | | |
| **Variable** | **Estimate** | **Std. Error** | **z value** | **Pr(>\|z\|)** | **Signif.** | **Odds Ratio (OR)** | **95% CI (Lower)** | **95% CI (Upper)** |
| **(Intercept)** | -3.019 | 0.136 | -22.175 | 6.01E‑109 | *** | 0.05 | 0.04 | 0.06 |
| **pct_black_or_african_american** | 0.037 | 0.003 | 12.858 | 7.73E-38 | *** | 1.04 | 1.03 | 1.04 |
| **pct_other_limited_english_household** | 0.247 | 0.085 | 2.904 | 3.69E-03 | ** | 1.28 | 1.08 | 1.51 |
| **SEF_2** | 13.708 | 4.007 | 3.421 | 6.25E-04 | *** | 8.98E+05 | 348.42 | 2.31E+09 |
| **SEF_4** | 17.714 | 3.525 | 5.026 | 5.01E-07 | *** | 4.92E+07 | 49304.6 | 4.94E+10 |
| **SEF_7** | 24.684 | 4.716 | 5.234 | 1.66E-07 | *** | 5.24E+10 | 5.08E+06 | 5.43E+14 |
| **SEF_12** | -16.45 | 3.512 | -4.684 | 2.81E-06 | *** | 0 | 0 | 0 |

**S5 Table, Period 4.** Final Logistic Regression Models for Period 4 (December 2020 - February 2021). Variables with SEF_ prefix represent spatial eigenvectors to account for spatial dependence

| **Period 4: December 2020 - February 2021** | | | | | | | | |
| --- | --- | --- | --- | --- | --- | --- | --- | --- |
| **Logistic regression results predicting if a block group belongs to a hot spot** | | | | | | | | |
| **Variable** | **Estimate** | **Std. Error** | **z value** | **Pr(>\|z\|)** | **Signif.** | **Odds Ratio (OR)** | **95% CI (Lower)** | **95% CI (Upper)** |
| **(Intercept)** | -2.14 | 0.31 | -6.908 | 4.93E-12 | *** | 0.12 | 0.06 | 0.22 |
| **pct_two_plus_races** | -0.032 | 0.011 | -3.03 | 2.45E-03 | ** | 0.97 | 0.95 | 0.99 |
| **pct_walked** | 0.064 | 0.018 | 3.593 | 3.27E-04 | *** | 1.07 | 1.03 | 1.1 |
| **pct_family_households_other_family** | -0.037 | 0.009 | -4.017 | 5.89E-05 | *** | 0.96 | 0.95 | 0.98 |
| **SEF_5** | -45.476 | 6.991 | -6.505 | 7.77E-11 | *** | 0 | 0 | 0 |
| **SEF_8** | 20.391 | 6.027 | 3.383 | 7.16E-04 | *** | 7.17E+08 | 5313.61 | 9.66E+13 |
| **SEF_9** | 28.499 | 6.381 | 4.466 | 7.97E-06 | *** | 2.38E+12 | 8.81E+06 | 6.46E+17 |
| **SEF_12** | -24.005 | 5.127 | -4.682 | 2.84E-06 | *** | 0 | 0 | 0 |
|  |  |  |  |  |  |  |  |  |
| **Logistic regression results predicting if a block group belongs to a cold spot** | | | | | | | | |
| **Variable** | **Estimate** | **Std. Error** | **z value** | **Pr(>\|z\|)** | **Signif.** | **Odds Ratio (OR)** | **95% CI (Lower)** | **95% CI (Upper)** |
| **(Intercept)** | -2.884 | 0.129 | -22.322 | 2.24E‑110 | *** | 0.06 | 0.04 | 0.07 |
| **pct_black_or_african_american** | 0.021 | 0.003 | 6.475 | 9.48E-11 | *** | 1.02 | 1.01 | 1.03 |
| **pct_other_indo_european_limited_english_household** | 0.052 | 0.016 | 3.32 | 8.99E-04 | *** | 1.05 | 1.02 | 1.09 |
| **pct_other_limited_english_household** | 0.291 | 0.086 | 3.371 | 7.50E-04 | *** | 1.34 | 1.13 | 1.58 |
| **SEF_2** | 13.314 | 4.302 | 3.095 | 1.97E-03 | ** | 6.04E+05 | 132.05 | 2.78E+09 |
| **SEF_4** | 11.192 | 3.928 | 2.849 | 4.38E-03 | ** | 72520 | 32.88 | 1.60E+08 |
| **SEF_7** | 15.592 | 4.84 | 3.221 | 1.28E-03 | ** | 5.89E+06 | 448.26 | 7.79E+10 |
| **SEF_8** | 10.409 | 3.484 | 2.987 | 2.81E-03 | ** | 33150 | 35.87 | 30660000 |
| **SEF_12** | -12.525 | 3.724 | -3.364 | 7.69E-04 | *** | 0 | 0 | 0 |

**S5 Table, Period 5.** Final Logistic Regression Models for Period 5 (March 2021 - May 2021). Variables with SEF_ prefix represent spatial eigenvectors to account for spatial dependence

| **Period 5: March 2021 - May 2021** | | | | | | | | |
| --- | --- | --- | --- | --- | --- | --- | --- | --- |
|  |  |  |  |  |  |  |  |  |
| **Logistic regression results predicting if a block group belongs to a hot spot** | | | | | | | | |
| **Variable** | **Estimate** | **Std. Error** | **z value** | **Pr(>\|z\|)** | **Signif.** | **Odds Ratio (OR)** | **95% CI (Lower)** | **95% CI (Upper)** |
| **(Intercept)** | -2.076 | 0.293 | -7.073 | 1.51E-12 | *** | 0.13 | 0.07 | 0.22 |
| **pct_black_or_african_american** | -0.018 | 0.007 | -2.687 | 7.21E-03 | ** | 0.98 | 0.97 | 1 |
| **pct_some_other_race** | -0.06 | 0.023 | -2.623 | 8.71E-03 | ** | 0.94 | 0.9 | 0.98 |
| **pct_two_plus_races** | -0.046 | 0.012 | -3.776 | 1.59E-04 | *** | 0.96 | 0.93 | 0.98 |
| **SEF_4** | 23.737 | 5.795 | 4.096 | 4.20E-05 | *** | 2.04E+10 | 2.38E+05 | 1.58E+15 |
| **SEF_5** | -19.475 | 6.193 | -3.145 | 1.66E-03 | ** | 0 | 0 | 0 |
| **SEF_9** | 21.142 | 6.999 | 3.021 | 2.52E-03 | ** | 1.52E+09 | 1674.8 | 1.38E+15 |
| **SEF_12** | -17.233 | 5.47 | -3.15 | 1.63E-03 | ** | 0 | 0 | 0 |
|  |  |  |  |  |  |  |  |  |
| **Logistic regression results predicting if a block group belongs to a cold spot** | | | | | | | | |
| **Variable** | **Estimate** | **Std. Error** | **z value** | **Pr(>\|z\|)** | **Signif.** | **Odds Ratio (OR)** | **95% CI (Lower)** | **95% CI (Upper)** |
| **(Intercept)** | -2.908 | 0.133 | -21.868 | 5.24E‑106 | *** | 0.05 | 0.04 | 0.07 |
| **pct_black_or_african_american** | 0.012 | 0.004 | 3.347 | 8.17E-04 | *** | 1.01 | 1.01 | 1.02 |
| **pct_other_indo_european_limited_english_household** | 0.061 | 0.015 | 4.096 | 4.21E-05 | *** | 1.06 | 1.03 | 1.09 |
| **SEF_4** | 14.579 | 3.976 | 3.667 | 2.45E-04 | *** | 2.14E+06 | 885.9 | 5.19E+09 |
| **SEF_9** | 13.76 | 5.136 | 2.679 | 7.38E-03 | ** | 9.46E+05 | 40.21 | 2.23E+10 |
