# Supplementary material for: The detection paradox: How COVID-19 surveillance patterns shifted from lower-income to more affluent neighborhoods in Miami-Dade County
Source: PLoS One. 2026 Oct 23;21(10):e0361211. doi: 10.1371/journal.pone.0361211 (PMC13693879; doi:10.1371/journal.pone.0361211)
Supplement: Supplementary file 8 [file pone.0361211.s008.docx]

**S1 File. Supplementary Methods**

The following model specifications are referenced in the main-text Methods, under Geospatial analysis. Variable definitions follow each equation below.

**Eq 1.** Local Getis-Ord Gi* statistic used for hot- and cold-spot detection (Hot spot analysis).

$$G_{i}^{*}=\frac{\sum_{j=1}^{n} w_{i,j}x_{j}-\bar{X}\sum_{j=1}^{n} w_{i,j}}{S\sqrt{\frac{n\sum_{j=1}^{n} w_{i,j}^{2}-\left( \sum_{j=1}^{n} w_{i,j} \right)^{2}}{n-1}}}$$

where *x_j_* is the value of the variable of interest (i.e. the COVID-19 infection rate in a study period) at block group *j*, *n* is the number of block groups, *w_i,j_* is the spatial weight between locations *i* and *j*, *X̄* is the global mean, and *S* is the global standard deviation.

**Eq 2.** Negative binomial regression model of COVID-19 case counts (Spatial regression modeling with spatial eigenvector filtering).

$$log(\mu_{i})=\beta_{0}+\beta_{1}X_{1i}+\beta_{2}X_{2i}+...+\beta_{k}X_{ki}+log(pop_{i})$$

where *μ_i_* is the expected count of COVID-19 cases in block group *i*, *X_1i_,X_2i_,...,X_ki_* represent the socio-economic, control, and spatial filter variables for block group *i*, *β_0_* is the intercept, *β_1_,β_2_,...β_k_* are the regression coefficients, and *log(pop_i_)* is the offset.

**Eq 3.** Logistic regression model of spatial cluster membership (Classification of cluster membership).

$$\log\left( \frac{P\left( Y_{i}=1 \right)}{1-P\left( Y_{i}=1 \right)} \right)=\beta_{0}+\beta_{1}X_{1i}+\beta_{2}X_{2i}+\cdots+\beta_{k}X_{ki}$$

where *P(Y_i_=1)* is the probability that block group *i* belongs to a Hot Spot or Cold Spot cluster, *X_1i_, X_2i_, …, X_ki_* represent socio-economic and control variables for block group *i*, *β_0_* is the intercept, and *β_1_,β_2_,...,β_k_* are the regression coefficients.
