# Supplementary material for: The detection paradox: How COVID-19 surveillance patterns shifted from lower-income to more affluent neighborhoods in Miami-Dade County
Source: PLoS One. 2026 Oct 23;21(10):e0361211. doi: 10.1371/journal.pone.0361211 (PMC13693879; doi:10.1371/journal.pone.0361211)
Supplement: Supplementary file 9 [file pone.0361211.s009.docx]

STROBE Statement—Checklist of items that should be included in reports of ***cross-sectional studies***

|  | Item No | Recommendation | Reported in |
| --- | --- | --- | --- |
| **Title and abstract** | 1 | (*a*) Indicate the study’s design with a commonly used term in the title or the abstract | Title; Abstract |
|  |  | (*b*) Provide in the abstract an informative and balanced summary of what was done and what was found | Abstract |
| Introduction | | | |
| Background/rationale | 2 | Explain the scientific background and rationale for the investigation being reported | Introduction |
| Objectives | 3 | State specific objectives, including any prespecified hypotheses | Introduction |
| Methods | | | |
| Study design | 4 | Present key elements of study design early in the paper | Methods |
| Setting | 5 | Describe the setting, locations, and relevant dates, including periods of recruitment, exposure, follow-up, and data collection | Methods, Study setting |
| Participants | 6 | (*a*) Give the eligibility criteria, and the sources and methods of selection of participants | Not applicable as written. The design is ecological. The units of analysis are census block groups, not individuals. Their definition, source, and inclusion criteria are given in Methods, Study setting and Data sources. |
| Variables | 7 | Clearly define all outcomes, exposures, predictors, potential confounders, and effect modifiers. Give diagnostic criteria, if applicable | Methods, Data sources through CBG socioeconomic measures |
| Data sources/ measurement | 8* | For each variable of interest, give sources of data and details of methods of assessment (measurement). Describe comparability of assessment methods if there is more than one group | Methods, Data sources |
| Bias | 9 | Describe any efforts to address potential sources of bias | Methods, Data sources; Methods, Geospatial analysis; Discussion, Limitations and Future Directions. Differential case ascertainment is the subject of the study. The surveillance address field carried no indicator of its source, so testing site locations could not be modeled, and this is stated as a limitation. |
| Study size | 10 | Explain how the study size was arrived at | Not applicable as written. No sample was drawn. All 1,831 block groups inside the Urban Development Boundary were included (Methods, Study setting). The regression models were fit on the block groups with complete covariate data. |
| Quantitative variables | 11 | Explain how quantitative variables were handled in the analyses. If applicable, describe which groupings were chosen and why | Methods, Data sources through Geospatial analysis |
| Statistical methods | 12 | (*a*) Describe all statistical methods, including those used to control for confounding | Methods, Geospatial analysis |
|  |  | (*b*) Describe any methods used to examine subgroups and interactions | Methods, Geospatial analysis |
|  |  | (*c*) Explain how missing data were addressed | Methods. Median household income was unavailable for 203 block groups and reported as US$250,000 or more, the ACS top code, for 20 others. Both were treated as missing, so the regression models were fit by complete-case analysis on 1,608 block groups, while the Getis-Ord Gi* analysis used all 1,831. The same analytic set was used for every regression model. |
|  |  | (*d*) If applicable, describe analytical methods taking account of sampling strategy | N/A |
|  |  | (*e*) Describe any sensitivity analyses | N/A |
| Results | | | |
| Participants | 13* | (a) Report numbers of individuals at each stage of study—eg numbers potentially eligible, examined for eligibility, confirmed eligible, included in the study, completing follow-up, and analysed | Not applicable as written. No individuals were enrolled or followed. Case counts and the number of block groups analyzed are given in Results, Descriptive statistics. |
|  |  | (b) Give reasons for non-participation at each stage | N/A |
|  |  | (c) Consider use of a flow diagram | N/A |
| Descriptive data | 14* | (a) Give characteristics of study participants (eg demographic, clinical, social) and information on exposures and potential confounders | Results, Descriptive statistics; Table 2; Table 3. Characteristics describe block groups rather than individuals. |
|  |  | (b) Indicate number of participants with missing data for each variable of interest | A numeric median household income was unavailable for 223 of 1,831 block groups, 203 with no value reported and 20 top-coded at US$250,000 or more. No other covariate was missing for more than 11. |
| Outcome data | 15* | Report numbers of outcome events or summary measures | Results, Descriptive statistics; Factors associated with overall COVID-19 rates |
| Main results | 16 | (*a*) Give unadjusted estimates and, if applicable, confounder-adjusted estimates and their precision (eg, 95% confidence interval). Make clear which confounders were adjusted for and why they were included | Results, Factors associated with overall COVID-19 rates through Socio-demographic Predictors; Fig 2; Fig 5 |
|  |  | (*b*) Report category boundaries when continuous variables were categorized | N/A |
|  |  | (*c*) If relevant, consider translating estimates of relative risk into absolute risk for a meaningful time period | N/A |
| Other analyses | 17 | Report other analyses done—eg analyses of subgroups and interactions, and sensitivity analyses | Results, Socio-Demographic Profiles; Socio-demographic Predictors |
| Discussion | | | |
| Key results | 18 | Summarise key results with reference to study objectives | Discussion, The Testing Access Paradox |
| Limitations | 19 | Discuss limitations of the study, taking into account sources of potential bias or imprecision. Discuss both direction and magnitude of any potential bias | Discussion, Limitations and Future Directions |
| Interpretation | 20 | Give a cautious overall interpretation of results considering objectives, limitations, multiplicity of analyses, results from similar studies, and other relevant evidence | Discussion, The Testing Access Paradox through Rethinking Pandemic Preparedness |
| Generalisability | 21 | Discuss the generalisability (external validity) of the study results | Discussion |
| Other information | | | |
| Funding | 22 | Give the source of funding and the role of the funders for the present study and, if applicable, for the original study on which the present article is based | Funding statement (submission system) |

*Give information separately for exposed and unexposed groups.

**Note:** An Explanation and Elaboration article discusses each checklist item and gives methodological background and published examples of transparent reporting. The STROBE checklist is best used in conjunction with this article (freely available on the Web sites of PLoS Medicine at http://www.plosmedicine.org/, Annals of Internal Medicine at http://www.annals.org/, and Epidemiology at http://www.epidem.com/). Information on the STROBE Initiative is available at www.strobe-statement.org.
