# Supplementary material for: The detection paradox: How COVID-19 surveillance patterns shifted from lower-income to more affluent neighborhoods in Miami-Dade County
Source: PLoS One. 2026 Oct 23;21(10):e0361211. doi: 10.1371/journal.pone.0361211 (PMC13693879; doi:10.1371/journal.pone.0361211)
Supplement: Supplementary file 10 [file pone.0361211.s011.docx]

**Response to the Editor and Reviewer**

*Manuscript PONE-D-26-06257*

*The detection paradox: How COVID-19 surveillance patterns shifted from lower-income to more affluent neighborhoods in Miami-Dade County*

PLOS ONE

We thank the Academic Editor and Reviewer 1 for a careful and constructive reading of the manuscript. We are grateful for the positive assessment and for the specific suggestions, which have improved the paper. We revised it with three goals: to make the results easier to follow, to state the central argument more directly, and to meet each journal requirement. To improve readability we moved reference material to the Supporting Information, replaced the two dense regression summary tables with figures that show the temporal pattern at a glance, and reduced the number of large tables in the main text. To sharpen the message we revised the Introduction and Discussion so that the case for census block group analysis in surveillance and resource allocation is explicit. We also clarified that the five study periods serve as a temporal frame for the spatial analysis rather than a causal evaluation of policy, and we added a supporting reference.

In the responses below, the comments from the Editor and Reviewer appear in italics in a shaded bar. Our replies follow in plain text. Proposed manuscript wording appears in a blue box so it can be located and checked.

**1. Response to the Academic Editor**

**Editor comment on readability:**

*Although the topic is highly relevant and important, the manuscript is difficult to follow in its current form. Consider moving some tables to the Supplementary Material to improve readability and allow the key findings to stand out more clearly.*

**Response.** We agree that the main text carried too many large tables, and we reorganized it. The full list of 39 community-level variables, formerly Table 2, now appears in the Supporting Information, with a short description kept in the Methods. The three model equations, for the Getis-Ord Gi* statistic, the negative binomial regression, and the logistic regression, were moved to a new Supplementary Methods section, with a brief description of each method kept in the text. Figure 1, which orients the reader to the study area and the distribution of cases, was moved to the Supporting Information as well. After these moves the remaining tables and figures were renumbered, so items that the reviews refer to by their original numbers now carry new numbers, which we give in each response below.

We also replaced the two dense regression summary tables with figures. The negative binomial predictor summary is now Figure 2, and the logistic cluster-membership summary is now Figure 5, shown as two panels for the hot-spot and cold-spot models. Figure 2 encodes the direction of an association by color and the significance level by shading. Figure 5 uses triangle direction for the direction of the association and triangle size for the significance level. Both make the early-to-late shift immediate. The complete per-period estimates remain in the Supporting Information.

The main text now retains Table 1 for the policy context, Table 2 for the socio-economic summary statistics, Table 3 for the case counts by period, and Table 4 for the socio-demographic profiles of the hot and cold spots. The figures are Figure 1 for the case-count timeline, Figure 2 for the negative binomial predictor heatmap, Figure 3 for the hot- and cold-spot cluster maps, Figure 4 for the cold spots along the I-95 corridor, and Figure 5 for the logistic cluster-membership figure. These changes let the detection paradox and the temporal shift in cluster composition stand out.

**Editor comment on the logistic regression table (Table 7 in the original submission):**

*Table 7 is an important result but difficult to interpret and may benefit from simplification.*

**Response.** We agree, and we replaced the table with a figure. The table the Editor identified as Table 7 listed many variables across five periods and two cluster types, and most cells were blank, which buried the few strong and consistent signals. It is now Figure 5, a two-panel figure. The left panel is the hot-spot model and the right panel is the cold-spot model. Triangle direction shows the direction of each association, and triangle size shows the significance level. The figure is organized so the result is visible at once: in the early periods, income and household composition predicted cluster membership, and from Period 3 onward the proportion of Black residents became the dominant predictor of cold-spot membership. The full model output for every period and cluster type remains in the Supporting Information.

**2. Response to the Journal Requirements**

**Requirement 1. Style and file naming**

**Response.** We reviewed the manuscript against the PLOS ONE style templates for the main body and for the title page, authors, and affiliations. We adjusted the title page, the headings, and the reference formatting to match, and we named the submission files to the journal convention, with separate files for the Response to Reviewers, the Revised Manuscript with Track Changes, and the clean Manuscript.

**Requirement 2. Funding Information and Financial Disclosure must match**

**Response.** We reconciled the Funding Information and the Financial Disclosure so that they are identical. The award carries no grant number, so the grant number field in the submission system is left blank. Both now read as follows.

**Financial Disclosure (and matching Funding Information):** This work was supported by the Florida Blue Foundation (https://www.floridablue.com/foundation) through its COVID-19 Disaster Response program (award to D.R.B. and N.A.G.). The funder had no role in study design, data collection and analysis, the decision to publish, or preparation of the manuscript.

**Requirement 3. Ethics statement belongs in the Methods only**

**Response.** We moved the ethics statement into the Methods and removed it from every other location. The standalone Ethics and Informed Consent Statements section that previously appeared after the Conclusions has been deleted. The Methods now include the following subsection.

**Methods, Ethics approval subsection:** This study was conducted in accordance with the Declaration of Helsinki and applicable guidelines and regulations. It was approved by the Institutional Review Boards of the Florida Department of Health (IRB number 2022-012) and Florida International University (IRB number IRB-21-0081). The analysis used de-identified secondary data. The requirement for informed consent was waived because the dataset comprised all COVID-19 cases reported to the Florida Department of Health and individual consent was not practicable.

**Requirement 4. Map and satellite figures and the CC BY 4.0 license**

**Response.** We addressed the copyright requirement for each figure that contains map content. The analytical content in every figure, the block group boundaries, the case locations, the cluster classifications, and the spatial eigenvector values, was produced by the authors. Only the background reference layers raised the concern, and we handled them as follows.

Three figures contain map content: the hot- and cold-spot cluster maps (Figure 3), the I-95 corridor map (Figure 4), and the study-area map (S1 Fig, which was Figure 1 in the original submission and is now in the Supporting Information). We drew all three over public-domain base layers so they meet the CC BY 4.0 requirement. Block group boundaries, roads and highways, and water features are taken from the U.S. Census Bureau TIGER/Line files, which are public-domain works of the U.S. government, with the detailed shoreline taken from the Miami-Dade County Open Data Hub. The I-95 corridor map is drawn over public-domain orthoimagery from the U.S. Geological Survey. We updated each caption to identify the public-domain sources.

The full logistic regression results in the Supporting Information previously appeared as small reference maps over a satellite image alongside the regression tables. Those maps duplicated the cluster maps in the main text. We removed the maps and kept the regression tables, which are the unique content. This removes the satellite image from the Supporting Information without any loss of information.

The spatial eigenvector filter maps in the Supporting Information were drawn only from the public-domain Census TIGER/Line block group geometry and the authors' own computed values. They contain no proprietary base map and no satellite image, so they already meet the CC BY 4.0 requirement. We note this in case the original flag was applied to every map figure by default.

**Requirement 5. Recommended citations**

**Response.** The reviewer did not request any specific published work, and the decision letter recommends none; Requirement 5 is the journal's standard prompt. The reviewer's first comment asked us to support, with a reference, the point that the study periods cannot by themselves separate policy effects from the natural course of transmission. We added a reference for that point, described in our response to that comment below. No further citations are required.

**3. Response to Reviewer 1**

We thank Reviewer 1 for the positive assessment and for identifying the points that would make the paper clearer.

**Reviewer comment 1:**

*The most important issue is if a 3 month period of policies is enough or we are just seeing the natural transmission of the disease. Need to reference this.*

**Response.** This is an important point, and we addressed it in two ways. The study spans fifteen months, divided into five consecutive three-month periods, so the question is whether changes within those windows can be read as policy effects. First, we now state plainly that the study is descriptive and that the periods provide a temporal frame for organizing the spatial analysis, not a design for isolating the effect of any single policy. Second, we added a limitation that explains why an observational design over three-month windows cannot separate policy effects from the natural course of the epidemic, and we cite a study that makes this problem explicit. Stokes and colleagues, examining 130 countries, report that no established method cleanly attributes outcomes to multiple interventions introduced at the same time, which is the situation throughout our study period.

**Methods, Policy Context, added sentence:** These periods provide a temporal frame for organizing the spatial analysis. They are aligned with documented shifts in testing capacity and policy, but the study does not estimate the causal effect of any individual policy.

**Discussion, Limitations and Future Directions, added paragraph:** The five study periods were defined to align with documented shifts in testing capacity and public health policy, but the design is descriptive and does not isolate the effect of any single intervention. The changes in reported cases across periods reflect the combined influence of transmission dynamics, population behavior, testing availability, variant emergence, and policy, and these contributions cannot be separated within three-month observational windows. Behavior also changes voluntarily as case counts rise, independent of mandates, and no established method cleanly attributes outcomes to multiple interventions introduced at the same time. We therefore read the periods as a temporal frame for describing spatial patterns of detection, not as an evaluation of policy.

**New reference.** Stokes J, Turner AJ, Anselmi L, Morciano M, Hone T. The relative effects of non-pharmaceutical interventions on wave one Covid-19 mortality: natural experiment in 130 countries. BMC Public Health. 2022;22:1113. doi:10.1186/s12889-022-13546-6

**Reviewer comment 2:**

*The intro is long and does not really make clear the important point: CBGs should be used in the future of pandemic preparedness instead of zip code analysis. To be honest I would have preferred to see two articles, one that looks at CBGs (methods) and the other about social determinants. I think by having one paper the two messages lose force.*

**Response.** We agree that the Introduction was long and that the central argument was not stated early enough. We shortened the Introduction, mainly by condensing the material on transportation and mobility, and we state the argument directly near the start and again at the end of the section. In a final editing pass we removed repeated wording and unnecessary qualifiers, which shortened the section further without cutting content. The census block group is the more appropriate unit for COVID-19 surveillance and resource allocation because it resolves the within-neighborhood differences in income, race, and access to care that broader units average away. Most prior work in South Florida, including the ZIP-code analyses we build on, could not see these patterns, and we make that connection explicit.

On the suggestion to split the paper, we considered it and chose to keep a single article, which is also the form in which the Editor invited the revision. The two parts are not separable here. The finer geographic resolution and the spatial eigenvector filtering are what reveal the detection paradox. The method is not a separate contribution that stands on its own; it is what makes the substantive finding visible. Presented apart, a methods paper would lack its motivating result, and a social determinants paper would rest on an analysis the reader could not evaluate. We revised the framing so the method serves the central finding, which keeps both messages strong rather than dividing them. In the Discussion we added a paragraph drawing the cold spots and the affluent hot spots into a single gradient of testing access, so both arguments reach one conclusion rather than competing.

**Introduction, sharpened framing near the study aims:** Most COVID-19 analyses in this region have relied on ZIP code tabulation areas, which average over neighborhoods that differ sharply in income, race, and access to care. Building on that work, we argue that the census block group is the more appropriate unit for surveillance and resource allocation, because it resolves the within-ZIP heterogeneity that shapes both transmission and detection. Demonstrating this resolution, and showing what it reveals about who is counted during a pandemic, is a central aim of this study.

**Reviewer comment 3:**

*Many figures like figure 1, table 2 and formulas need to be moved to the appendix as they might distract readership.*

**Response.** We made these moves, described in our response to the Editor's first comment. Figure 1, Table 2, and the three model equations now appear in the Supporting Information, with a brief description of each method kept in the text. This leaves the main text with the tables and figures that carry the findings.

**Reviewer comment 4:**

*Table 5 is difficult to understand.*

**Response.** We agree, and we replaced the dense table with a figure. The table the reviewer identified as Table 5, the negative binomial predictor summary, packed every variable, period, direction, and significance level into one grid. It is now Figure 2, a heatmap in which color shows the direction of each association with reported case rates and shading shows the significance level, with the variables grouped so the early, middle, late, and persistent patterns are visible. The complete per-period estimates, with incidence rate ratios, confidence intervals, and diagnostics, remain in the Supporting Information. We replaced the logistic regression summary, the former Table 7, with a figure as well. It is now Figure 5, described in our response to the Editor above.

**4. Additional changes**

The reorganization the Editor and Reviewer asked for moved tables into the Supporting Information and rebuilt two of them as figures. Reformatting that output gave us occasion to check the manuscript against the model results in full, not only the parts we moved. We compared every reported estimate with the model output.

Five values in the negative binomial results were transcribed incorrectly in the original submission, and we corrected them. The variable, period, direction, and significance of every association are unchanged. The corrected figures agree with Figure 2 and the estimates in the Supporting Information.

We also corrected two statements in the Methods about residual spatial autocorrelation. The baseline Moran's I values are significant at p < 0.05 rather than p < 0.001 in every period, and spatial eigenvector filtering reduced residual autocorrelation rather than removing it. The revised text reports the filtered range.

We also removed a claim the models do not support. The Abstract reported protective associations for household crowding and limited education. Overcrowding entered no final model, and lower educational attainment was associated with higher reported rates in the early periods. The Abstract now states the overcrowding result accurately, and two sentences in the Discussion and one in the Conclusions rest instead on renter occupancy, Black population share, and Medicaid enrollment. The Discussion now treats limited English proficiency as a predictor of cold spot membership, which the logistic models support.

Some additions were not prompted by the review. The study is ecological, and no reporting guideline covers this design directly. We followed the STROBE guideline for cross-sectional studies where its items apply, and the completed checklist is included as S1 Checklist. The items that assume individual participants, eligibility, and sampling are marked as not applicable, with the reason given. Working through the checklist also showed that the manuscript did not report its analytic sample. Median household income is unavailable for 203 block groups and reported as US$250,000 or more, the ACS top code, for 20 others. Both were treated as missing, so the regression models were fit on the 1,608 block groups with a numeric income value, while the Getis-Ord Gi* analysis used all 1,831. The same analytic set was used for every regression model, including those in which income was not retained after variable selection. The Methods now state this. We also checked what the 20 top-coded block groups would change. Recoding them to US$250,000 and refitting the logistic models on 1,628 block groups, with the model specification held fixed, left the direction and significance of the income association intact. The Period 2 result weakened from p < 0.001 to p < 0.01. The Methods and Results now report this check. We also corrected the permutation count reported for the Getis-Ord Gi* analysis. The Methods now report 499 permutations, the number used in the analysis, in place of 999. Cluster classification is unchanged. The Methods also describe the neighbor definition and the weights matrix, and note that the surveillance records carried a single address field with no indicator of its source, so testing site locations could not be modeled. The Limitations note this constraint and add a paragraph on generalizability.

We revised the title to “The detection paradox: How COVID-19 surveillance patterns shifted from lower-income to more affluent neighborhoods in Miami-Dade County,” to match the Abstract. Affiliations now reflect the institutions at which each author contributed. Following PLOS policy, the funding, competing interests, and author contribution statements are removed from the manuscript and entered in the submission system.

We thank the Editor and Reviewer for their time and for comments that improved the paper.
